# A Single-Arm, Proof-Of-Concept Trial of Lopimune (Lopinavir/Ritonavir) as a Treatment for HPV-Related Pre-Invasive Cervical Disease

**DOI:** 10.1371/journal.pone.0147917

**Published:** 2016-01-29

**Authors:** Lynne Hampson, Innocent O. Maranga, Millicent S. Masinde, Anthony W. Oliver, Gavin Batman, Xiaotong He, Minaxi Desai, Parmenas M. Okemwa, Helen Stringfellow, Pierre Martin-Hirsch, Alex M. Mwaniki, Peter Gichangi, Ian N. Hampson

**Affiliations:** 1 University of Manchester Viral Oncology Laboratories, Institute of Cancer Sciences, Research Floor 5, St Mary’s Hospital, Oxford Road, Manchester M13 9WL, United Kingdom; 2 Kenyatta National Hospital, Department of Reproductive Health, PO Box 20723–00202, Nairobi, Kenya; 3 University of Nairobi, College of Health Sciences, Departments of Gynaecology and Pathology, PO Box 19676–00202, Nairobi, Kenya; 4 Cytology Laboratories, PO Box 208, Clinical Sciences Building 2, Central Manchester University Hospital NHS Trust, Oxford Rd, Manchester M13 9WW, United Kingdom; 5 Department of Pathology, Royal Preston Hospital, Sharoe Green Lane, Fulwood, Preston, PR2 9HT, United Kingdom; 6 Department of Obstetrics & Gynaecology, Royal Preston Hospital, Sharoe Green Lane, Fulwood, Preston, PR2 9HT, United Kingdom; National Cancer Institute, UNITED STATES

## Abstract

**Background:**

Cervical cancer is the most common female malignancy in the developing nations and the third most common cancer in women globally. An effective, inexpensive and self-applied topical treatment would be an ideal solution for treatment of screen-detected, pre-invasive cervical disease in low resource settings.

**Methods:**

Between 01/03/2013 and 01/08/2013, women attending Kenyatta National Hospital's Family Planning and Gynaecology Outpatients clinics were tested for HIV, HPV (Cervista®) and liquid based cervical cytology (LBC -ThinPrep®). HIV negative women diagnosed as high-risk HPV positive with high grade squamous intraepithelial lesions (HSIL) were examined by colposcopy and given a 2 week course of 1 capsule of Lopimune (CIPLA) twice daily, to be self-applied as a vaginal pessary. Colposcopy, HPV testing and LBC were repeated at 4 and 12 weeks post-start of treatment with a final punch biopsy at 3 months for histology. Primary outcome measures were acceptability of treatment with efficacy as a secondary consideration.

**Results:**

A total of 23 women with HSIL were treated with Lopimune during which time no adverse reactions were reported. A maximum concentration of 10 ng/ml of lopinavir was detected in patient plasma 1 week after starting treatment. HPV was no longer detected in 12/23 (52.2%, 95%CI: 30.6–73.2%). Post-treatment cytology at 12 weeks on women with HSIL, showed 14/22 (63.6%, 95%CI: 40.6–82.8%) had no dysplasia and 4/22 (18.2%, 95%CI: 9.9–65.1%) were now low grade demonstrating a combined positive response in 81.8% of women of which 77.8% was confirmed by histology. These data are supported by colposcopic images, which show regression of cervical lesions.

**Conclusions:**

These results demonstrate the potential of Lopimune as a self-applied therapy for HPV infection and related cervical lesions. Since there were no serious adverse events or detectable post-treatment morbidity, this study indicates that further trials are clearly justified to define optimal regimes and the overall benefit of this therapy.

**Trial Registration:**

ISRCTN Registry 48776874

## Introduction

Infection with high-risk types of HPV has now been established as the main aetiological agent for invasive cervical cancer (ICC)[1,2,3] and globally there are >270,000 deaths from this disease per annum with over 85% of these occurring in low resource countries[4]. In Kenya, the setting for our trial, cervical cancer accounts for 23% of all female malignancies [5].

The development of ICC can take 10–20 years and is preceded by HPV related pre-invasive pathology which are characterised as either low-grade (CIN1) or high-grade cervical intraepithelial neoplasia (CIN2/3)[1]. These lesions can be screen detected by cervical cytology testing where they are diagnosed as either borderline atypical squamous cells of undetermined significance (ASCUS), low-grade squamous intraepithelial lesions (LSIL) or high-grade squamous intraepithelial lesions (HSIL)[6].

The reduction in ICC related mortality in the developed world has been largely dependent on organised cytology screening and similar trends in cervical cancer mortality have been achieved by organised single screen and treatment in low resource settings [7]. However, in the majority of these areas of the world, lack of resources and health education means that most pre-invasive cervical disease remains undiagnosed and untreated. Thus, where funds are limited, low-cost screening and treatment options are clearly a high priority.

Current treatment options in clinical practice are either by ablative (destructive) or excisional modalities which have similar success rates but have different morbidities [8]. In the developed nations, Large Loop Excision of the Transformation Zone (LLETZ) or Loop Electrosurgical Excision Procedure (LEEP) [9] is used in the majority of colposcopy clinics. Although highly effective, this procedure is associated with a risk of primary/secondary haemorrhage, prolonged vaginal discharge, infection and a risk of preterm delivery in subsequent pregnancies, which can be problematic in low resource countries. Ablative cold coagulation and cryotherapy are often advocated for use in these settings although some studies have suggested that these treatments have a higher failure rate when compared to other modalities [8,10].

There are a variety of locally-applied, non-surgical approaches which have been evaluated for the treatment of cervical dysplasia (Reviewed by Stern et al [11] and Bernard [12]). However, although some of these alternative treatments show promise, their outcomes are generally inferior to the reported 80–95% success rates obtained with ablative/excisional modalities when these are carried out in quality assured colposcopy units.

We originally speculated that HIV protease inhibitors (PIs) may have activity against HPV related cervical disease and identified lopinavir as the most effective [13] although this was at ten times the concentration achieved by oral dosing for HIV therapy [14]. Indeed, recent work has shown that oral lopinavir, as used for normal HIV therapy, has little effect on HPV status in HIV positive women [15]. Subsequent in vitro studies defined at least part of the mode-of-action of lopinavir against HPV [16] and many HIV PI’s are now well known to have more general anticancer properties [17,18,19]. Most notably, it has been suggested that ritonavir (usually co-administred with lopinavir as Lopimune or Kaletra) may have anti-invasive and anti-proliferative activity against cells derived from pre-invasive CINs but little activity against cells derived from more advanced ICC [20].

Based on our pre-clinical studies it was concluded that direct application of lopinavir to the cervix as a vaginal pessary or gel, might achieve the concentration required for activity against HPV related cervical dysplasia. Since we already had research collaborations in place with clinicians at Kenyatta National Hospital/University of Nairobi in Kenya, we built on these to conduct a trial of self-applied lopinavir (in the form of Lopimune) in this setting with the aim of developing an effective, inexpensive, non-surgical, self-applied treatment with minimal side effects. We now report the results of a small, single-arm study to evaluate the safety, acceptability and ability of Lopimune to induce clearance of HPV infection, and related dysplasia, in a group of 23 women diagnosed with HSIL.

## Methods

### Patients and Study Centres

This was a collaborative proof-of-concept clinical trial between the University of Manchester and the University of Nairobi/Kenyatta National Hospital (KNH), Kenya. Clinical and basic diagnostic tests were done at KNH while scientific aspects of the study that required specialized equipment were carried out at the Viral Oncology Laboratory, St Marys Hospital in Manchester, UK. KNH and the University of Nairobi Ethics and Research Committee (KNH/UoN-ERC) approved the trial on the 25/11/2011, an extension was granted on the 07/01/2013 and the trial was conducted between 01/03/2013 and 01/11/2013. Patients were recruited from women attending the Family Planning and Gynaecology Outpatient clinic at KNH for routine follow up. Registration was carried out retrospectively (ISRCTN48776874) since the original intention was to recruit a small number of patients to investigate acceptability of treatment.

### Enrolment and Procedures

**Fig 1:**
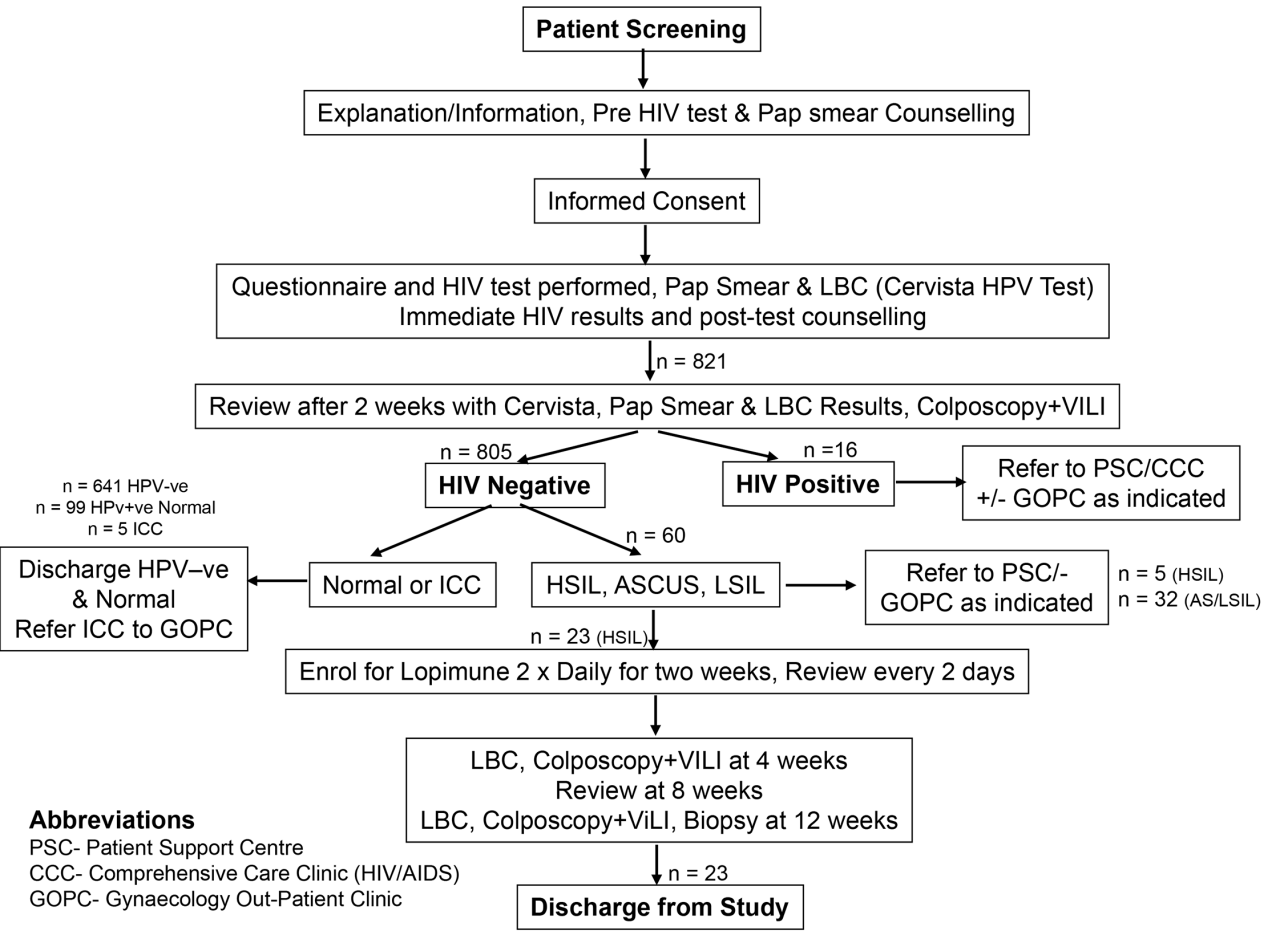
Patient screening, testing and management flow chart used for the study.

The study protocol is illustrated in Fig 1. At the screening stage, potential participants were given the patient information sheet counselled appropriately and those willing to participate gave informed signed consent. Thereafter, a structured questionnaire was used to take both the socio-demographic, sexual histories and clinical characteristics (S2 Text), Blood was drawn for a HIV test using Determine^TM^ (Abbott, USA) and if positive was confirmed by Unigold^TM^ (Trinity Biotech, Ireland) as per Kenya HIV testing algorithm. All patients were then given a speculum examination during which two cervical cyto-brush samples were taken. The first of these was used for LBC ThinPrep® and HPV testing and samples were immediately sent to Manchester. The second was used for a conventional smear test as per standard Pap smear screening procedures, which was examined and reported in Nairobi by the Kenyan study pathologist. Patients were then reviewed after one month at which time those that were HIV negative and HPV positive with abnormal cervical cytology, were enrolled into the Lopimune trial. It was decided this should be pragmatic in nature and biopsies were not taken at this point since it is well known this can affect treatment outcomes [21]. Furthermore it was also deemed important to avoid biopsy related bleeding or discharge since this would deter women from attendng for follow-up assessments. Consequently two independent cytology slides (Conventional reported by MPO and LBC reported by MD) were used to assess pre-treatment disease. HPV testing in combination with cytology and histology was used at the end of the study to determine post-treatment outcomes.

At enrolment a pelvic examination was carried out which included baseline colposcopy. Cervical morphology was initially visualised with acetic acid (VIA) followed by Lugol’s Iodine (VILI) using x5 magnification (Video Colposcope, Welch Allyn. NY. USA). This was carried out according to standard clinical practices and images recorded [22]. Blood was drawn for a full blood count, urea, creatinine, electrolytes and liver function tests. Each patient was then issued with a supply of Lopimune soft gel capsules (133 mg lopinavir/33 mg ritonavir, CIPLA, India) for vaginal insertion of one capsule once or twice daily for a maximum of 2 weeks with the intention of block radomising the dose. Two additional visits per week were scheduled for the two-week duration of Lopimune therapy during which a structured questionnaire was filled out and a physical examination performed, both of which were conducted by either the Nairobi PI (IOM) or study doctor (Dr Masinde, Resident in Gynaecology, University of Nairobi) in order to assess occurrence of adverse drug reactions and drug compliance (S3 Text and S4 Text). Enrolled patients also had serial visits scheduled at 1, 2, 4, 8, 12 and 16 weeks. Follow up LBC ThinPrep® samples were taken at 4 and 12 weeks and sent to Manchester for cytology and HPV testing. In addition, colposcopy with VILI was carried out at 4 and 12 weeks and final punch biopsies were taken at the latter time point. Blood was also drawn at these visits for baseline clinical tests described previously. The 8-week visit was to assess any potential drug reactions and to remind women that a punch biopsy would be taken from any abnormal areas detected by VIA or VILI on the cervix at the 12-week visit. Biopsies were stored in 10% buffered formalin and were sent to Manchester. Those patients diagnosed with post-treatment, HPV positive high-grade disease were referred for Loop electrosurgical excision procedure (LEEP). These were reviewed again at 5 months and advised to continue routine Pap smear screening at 6 months thereafter.

### Cervista® HR-HPV Test

All ThinPrep® LBC samples sent to Manchester were analysed by staff trained and certified by Hologic (Hologic, Bedford, MA). HPV testing of LBC samples was carried out using the FDA-approved Cervista HPV HR test [23] in conjunction with the Cervista MTA (Hologic) automated platform according to the manufacturer’s instructions. Prior to and after analysis, all LBC samples were stored at +4°C in a monitored refrigerator.

### HPV Genotyping

Ultrapure DNA isolated from each ThinPrep® LBC sample which was used for the Cervista® test, was then further analysed by PCR to test for which HPV genotypes were present in each respective Cervista® mastermix positive sample. A novel hot-start, touch-down multiplex method was used which simultaneously detects the L1, E6 and E7 ORFs of HPV type 16, 18, 31, 33, 35, 39, 45, 51, 52, 56, 58, 59, 66, 68, and 70 as previously described [24]. Each assay was repeated a minimum of three times.

### LBC ThinPrep® Cervical Cytology

LBC slide preparation was carried out using the ThinPrep-2000 system (Hologic) automated slide preparation unit, according to the manufactures directions and Pap stained (Hologic) at the Regional Cytology Laboratories (Clinical Sciences Building, Manchester Royal Infirmary, UK). Reporting of the LBC samples was done using a Dual Review Imaging System (Hologic).

### Cervical Pathology

Final punch biopsy samples of the cervical transformation zone and VILI positive lesions were transported to Manchester, wax embedded and 3–4μm sections cut at 3 levels according to standard pathology laboratory practices. The sections were then stained with haematoxylin and eosin and reported separately by two pathologists Independent review of cases where there was disagreement was carried out by a third pathologist (Dr Bianca Da Gama Rose).

### Analysis of Lopinavir Concentration in Plasma

Lopinavir was extracted from plasma using a method based on that of Djerada et al [25]. MSMS analysis was carried out by use of an Agilent 6520 QTOF: 150-1000m/z targeted MSMS 629.37 between 2.5–5.5 min (50–800 m/z). Standard amounts of ultrapure research grade lopinavir (Santa Cruz Biotechnology, Inc. Heidelberg, GMBH) comprising 1000, 500, 100, 50, 25, 12.5 and 6.25ng were added to control plasma and extracted as above. All test plasma extractions and assays were performed in triplicate and were assayed with lopinavir control standards.

### Statistical Methods

Agreements between the conventional and ThinPrep cytology and the two study histopathologists were analysed by the McNemar test using R program version 3.1.2. All 95% confidence intervals were computed using the R binom package and a p value < 0.05 was considered statistically significant.

## Results

All baseline bloods and health checks were normal for the study participants.

### Primary HPV and Cytology Screening

Fig 1 shows the management scheme for the 821 women screened of which 16 were HIV positive. Out of the remaining 805 HIV negative women, 164 (20.4%, 95%CI: 17.6–23.3%) were Cervista® positive for high-risk HPV. Out of these, cytology showed that 99 (60.3%, 95%CI: 52.5–67.9) were normal, 28 (17.1%, 95%CI: 11.7–23.7) had HSIL, 11 (6.7%, 95%CI: 3.4) had LSIL and 21 (12.8%, 95%CI: 8.1–18.9%) had ASCUS. Five (3.0%, 95%CI: 1.0–7.0%) were diagnosed with ICC and were sent for immediate biopsy and subsequent treatment (hysterectomy). Out of the 28 women identified with HSIL, 5 opted for alternative treatment with 23 eventually being enrolled on the trial.

### Lopimune Treatment of Women Diagnosed with HSIL

Table 1 shows the baseline characteristics of the 23 women diagnosed with HSIL who had an average mean age of 34.2 yrs prior to being recruited into the trial. Predictably, a combined relatively high incidence of HPV types 16 and 18 was detected which amounted to 10/23 (43.5%, 95%CI: 23.2–65.5%) of cases. With 5 infections, type 52 was the next most common followed by 4 infections each for types 35, 58 and 33. On one occasion Cervista® detected a positive for more than one master mix whereas PCR only detected one HPV genotype present which could be due to differences in sensitivity between these methods. A good concordance between HSIL diagnosed by LBC in Manchester and conventional smear testing carried out in Kenya was observed with only 3/23 disparities. McNemar test demonstrated a p-value of 0.32 which confirmed there was no significant difference between these two modalities.

**Table 1:** Participant characteristics at study baseline.

*Characteristics*	*Results at baseline n(% of 23)*
Age: Mean (SD)	35.4 years (±10.4)
Median (interquartile range)	37 years (27–41)
**Birth Control Method**	
• Condom	2 (8.6)
•Tubal ligation	1 (4.3)
•Depoprovera or Implant	5 (21.7)
•Intrauterine device	5 (21.7)
•None	9 (39.1)
•Post-menopausal	1 (4.3)
**Parity**	
•Nulliparous	3 (13.0)
•1–4 live births	17 (73.9)
•>4 live births	3 (13.0)
**HPV results**	
• Cervista Mix 1 only (M1) (HPV 51, 56, 66, or 70) positive	1 (4.3)
• Cervista Mix 2 only (M2) (HPV 18, 39, 45, 59, 68) positive	4 (17.4)
• Cervista Mix 3 only (M3) (HPV 16, 31, 33, 35, 52, 58) positive	14 (60.0)
• Cervista Mix 1 + Mix 3 positive	2 (8.6)
• Cervista Mix 2 + Mix 3 positive	2 (8.6)
**High-Risk HPV typing by PCR**	
• HPV16 only	2 (8.6)
• HPV16 + Non-HPV16 types	5 (21.7)
• Non-HPV16 types only (HPV18/31/33/35/39/45/51/52/56/58/59/68/70)	16 (69.6)
**Cytology Results: Liquid Based Cytology**	
• Moderate dyskaryosis: HSIL	10 (43.5)
• Severe dyskaryosis: HSIL	13 (56.5)
**Cytology Results: Conventional Cytology**	
• LSIL	1 (4.3)
• ASC-H with or without AGC	2 (8.6)
• HSIL	20 (87.0)

ASC-H abnormal squamous cells–cannot exclude HSIL; AGC abnormal glandular cells.

The original intention was to block randomise Lopimune treatment starting at the higher dose. However, due to the nature of recruitment being few patients spaced by long time intervals, it was noted that the twice-daily dose produced excellent cervical lesion clearance with no significant adverse effects. This prompted the decision to adopt an adaptive design and abandon the planned lower dose arm since no patient had been put on the lower dose at that time. The study, therefore, became a single-arm non-randomized trial.

### Adverse Reactions, Tolerability and Adherence to Treatment

In the 4 weeks post start-of-treatment there were no serious adverse reactions or drug events such as death, life-threatening events, hospitalization or disability observed. Additionally, none of the study subjects reported, vaginal dryness, bleeding/spotting, painful intercourse, palpitations, unusual weakness, vomiting, decreased appetite, diarrhoea, pale stools, itchy skin, skin rashes, increased thirst, increased frequency of urination, visual disturbance, chest pains, or jaundice. However, there were some minor side-effects (Table 2) but only nausea achieved statistical significance (p = 0.037). The commonest complaint by study subjects was headache (3/13%) followed by vaginal irritation (2/8.7%); nausea (2/8.7%); feeling faint/dizziness (2/8.7%), abnormal vaginal discharge (2/8.7%) and abdominal pains (1/4.3%). In total, 5/21.7% of study subjects experienced some form of minor complaint within the 1^st^ month of taking Lopimune as described.

**Table 2:** Symptoms experienced by study subjects within one month of starting Lopimune treatment.

	*Week 1*		*Week 2*		*Week 4*		*Total*		*p-value*
Patient complaints:	No.	%	No.	%	No.	%	No.	%	
Abnormal vaginal discharge	2	8.7	1	4.3	-	-	2	8.7	0.134
Vaginal irritation	2	8.7	-	-	1	4.3	2	8.7	0.617
Headaches	1	4.3	2	8.7	-	-	3	13.0	0.617
Nausea	2	8.7	-	-	-	-	2	8.7	0.037
Abdominal pains	-	-	1	4.3	-	-	1	4.3	0.803
**Total No. of patients affected**	**4**	**17.4**	**4**	**17.4**	**1**	**4.3**	**5**	**21.7**	

(Note:- Some patients had more than one symptom, n = 23)

Information on drug adherence was self-reporting and 10 (43.5%) of patients completed their treatment on schedule without skipping a dose. Out of the remaining 13, 7 (53.8%) missed once, 2 (15.4%) missed twice, 1 (7.6%) missed three times, 2 (15.4%) missed for times and one subject missed 5 consecutive doses. The commonest reason for skipping doses was interruption by menstrual flow (6/46.2%), forgot (5/38.5%) and the inconvenience of carrying refrigerated Lopimune whilst away from home (2/15.4%). Significantly, none of the subjects skipped due to side-effects, problems inserting pessaries or because their partner wanted intercourse. Indeed, despite being advised to abstain during the treatment period, 5 (21.7%) of patients still reported as having had intercourse at least once. Neither omitted doses nor engaging in sexual intercourse had any statistically significant effect on the observed treatment outcomes.

### Treatment outcome of Lopimune on HPV and related dysplasia

**Fig 2:**
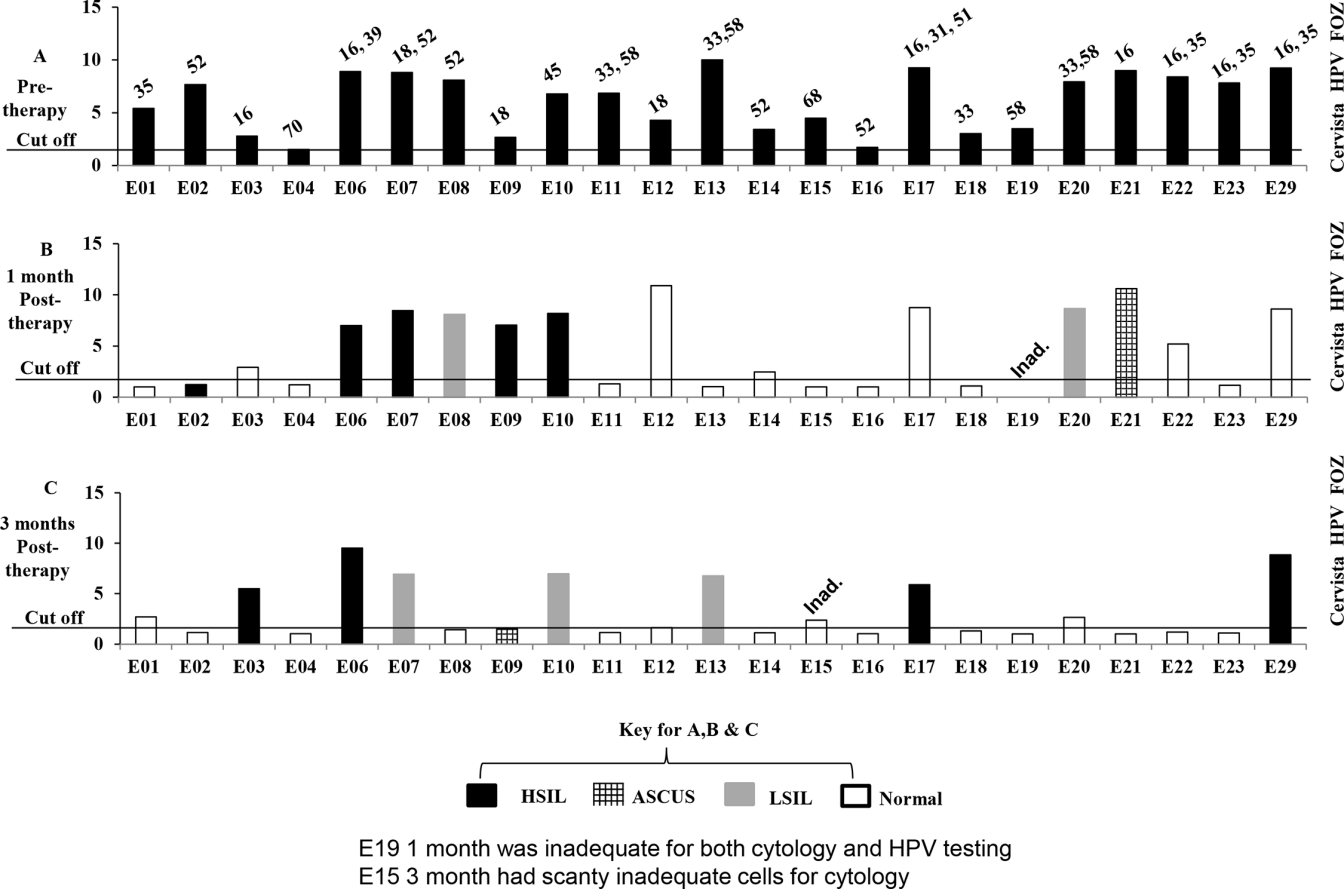
Cervista® HPV test, cytology and PCR HPV genotype analysis. HPV titre is indicated by the height of the bar graph above the cut off with the Y axis representing the fold over zero (FOZ) ratio of sample or control divided by the no target control signal. The cytology status is indicated by colour and shading with the HPV type shown above the bar. (A) Represents the results before treatment with lopinavir; (B) Represents results after 4 and (C) 12 weeks after treatment.

The results of repeat ThinPrep® LBC and Cervista® HPV tests carried out at 4 and 12 weeks post-start of treatment are summarised in Table 3 and Fig 2A, 2B and 2C. HPV genotypes present are shown above each bar of which the height indicates the level of HPV detected in each patient. This demonstrates that a post-treatment drop in the levels of HPV infection was observed in 19/23 (82.6%, 95%CI: 61.2–95.0%) with the virus not detected in 12/23 (52.2%, 95%CI: 30.6–73.2) of women 12 weeks after the start of treatment.

**Table 3:** Changes in cytology and HPV results between baseline and 3-month visits following Lopimune treatment.

Characteristics	Change between baseline and 3-months exit visit n(% of 23)
**Change in HPV results**	
• Any Cervista HPV-positive to Cervista HPV-negative	12 (52.2)
• Cervista Mix 1 only (M1) (HPV 51, 56, 66, or 70) positive to Cervista HPV-negative	1 (4.3)
• Cervista Mix 2 only (M2) (HPV 18, 39, 45, 59, 68) positive to Cervista HPV-negative	1 (4.3)
• Cervista Mix 3 only(M3) (HPV 16, 31, 33, 35, 52, 58) positive to Cervista HPV-negative	9 (39.1)
• Cervista Mix 1 + Mix 3	1 (4.3)
• Cervista Mix 2 + Mix 3	0 (0.0)
**Change in HPV16 and Non-HPV16 Types**	
• HPV16 only to HPV negative	1 (4.3)
• HPV16 + Non-HPV16 types	2 (8.6)
• Non-HPV16 high risk types (HPV18/31/33/35/39/45/51/52/56/58/59/68) to HPV negative	9 (39.1)
Change in liquid based cytology*	
• Any improvement in cytology	18 (81.8)
• HSIL to negative	14 (63.6)
○ Moderate dyskaryosis: HSIL to negative	5 (22.7)
○ Severe dyskaryosis: HSIL to negative	9 (40.9)
• HSIL to LSIL/ASCUS	4 (18.2)
○ Moderate dyskaryosis: HSIL to LSIL/ASCUS	3 (13.6)
○ Severe dyskaryosis: HSIL to LSIL/ASCUS	1 (4.5)

* Since one LBC sample was inadequate, percentages were calculated from n = 22.

A total of 22 patients were reported at 4 and 12 weeks since E19 had inadequate cellularity for HPV testing and cytology at 4 weeks whereas E15 was adequate for HPV testing but not cytology at 12 weeks. After 4 weeks (Fig 2B) 14/22 (63.6%, 95%CI: 40.6–82.8%) of patients had returned to normal cytology and 3/22 (13.6%, 95%CI: 2.91–34.9%) now had lower grade disease demonstrating a positive response in 76·9% of women. After 12 weeks (Fig 2C) 14/22 (63.6%, 95%CI: 40.6–82.8) of women still had normal cytology with 4/22 (18.2%, 95%CI: 5.2–40.3%) now presenting with lower grade dysplasia providing an overall positive response in 81.8% of women.

Comparison of histopathology results with cytology at 12 weeks showed that 13/22 (59.1%, 95%CI: 36.4–79.3%) had no detectable dysplasia and 4/22 (18.2%, 95%CI: 9.9–65.1%) had CIN1, which equates to a 77.3% positive response to treatment. Only 4/22 (18.2%, 95%CI: 9.9–65.1%) had CIN2 or CIN3 and 1/22 (4.5%, 95%CI: 0.1–22.9%) had hCGIN. A total of 22 patients were reported for pathology since the biopsies obtained from E19 at 12 weeks were inadequate and thus excluded from the analysis. McNemar test demonstrated a p-value of 0.025 which indicated that there was a statistically significant difference in reporting by the two study pathologists which was resolved by referral to a third independent pathologist.

### Colposcopy with VILI

**Fig 3:**
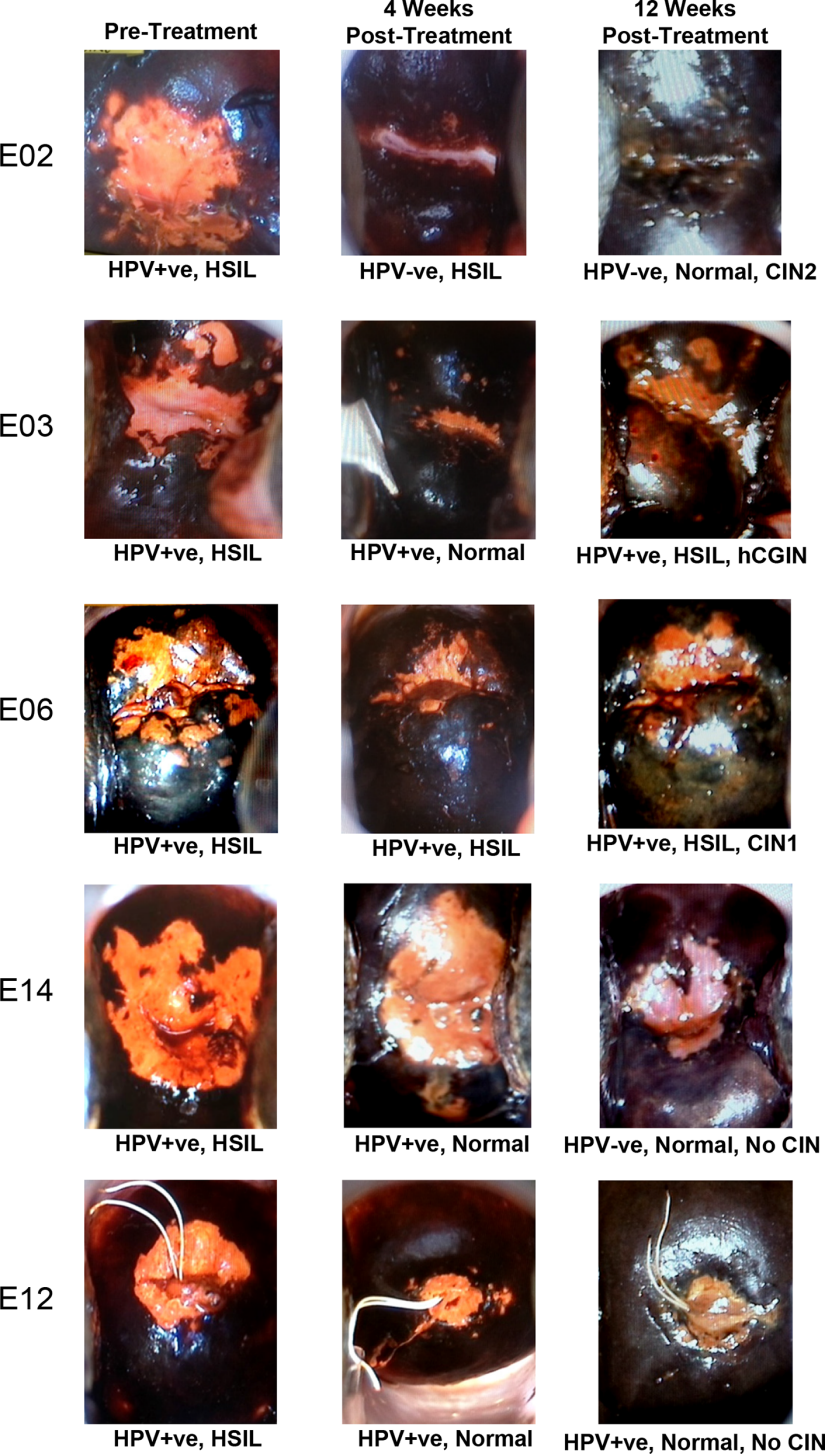
Colposcopy with VILI before, 4 weeks and 12 weeks after treatment for patients E02, E03, E06, E14 and E12. Magnification x5 was used with the Cervista® HPV status and cytology shown under each picture and final pathology (CIN status) also shown under the 12 weeks post-treatment images.

Fig 3 shows VILI colposcopic images of the cervix from 5 cases diagnosed with HSIL taken before, 4 and 12 weeks after treatment with Lopimune capsules. Extensive yellow/orange staining characteristic of poorly glycogenated dysplastic epithelium, was clearly present in all the pre-treatment images of the ectocervix and the cases shown were deliberately chosen to illustrate the types of response observed. The majority of patients showed a reduction in dysplastic epithelium, as detected by VILI, which was associated with reduced severity of disease as indicated by HPV, cytology and/or pathology status. For example patient E02 became HPV negative at 4 and 12 weeks and also clearly showed a marked regression of dysplastic epithelium detected by VILI although a small focus of CIN2 was still present. Patient E03 remained HPV positive at 4 and 12 weeks with normal cytology at 4 weeks and yet showed HSIL cytology with CIN1 and hCGIN at 12 weeks. E06 remained HPV positive with HSIL cytology after 12 weeks although pathology subsequently showed this to be CIN1. E14 and E12 both showed return to normal cytology at 4 and 12 weeks with no CIN present at 12 weeks although E12 remained HPV positive.

### Analysis of Plasma Levels of Lopinavir

Extracted ion chromatograms of research grade lopinavir showed this to have a mass/charge ratio of 629.37 m/z eluting at 5.1 min. Extraction of compound from control plasma was shown to be >90% efficient when compared to the same amount of drug added directly to solvent prior to analysis and could detect lopinavir to a level of ~6.00 ng/ml when added to plasma. Out of 23 samples tested, the maximum level of drug detected in patient plasma, after one week of vaginal delivery, was approximately 10 ng/ml although most samples were lower than this.

## Discussion

When used for oral HIV therapy, lopinavir is normally co-adminstered in a 4:1 ratio with ritonavir (Lopimune- CIPLA; Kaletra- AbbVie). Due to cost and supply issues, reformulation of lopinavir as a single agent was not possible so evaluation of the orally-administered Lopimune gelatine capsule formulation was undertaken with the axiom this might serve as a vaginal pessary for local delivery to the cervix. Clearly the local concentration of drug achieved by this mode of delivery will be much higher than the 74–215 ng/ml previously reported in cervico-vaginal fluid as a result of normal oral dosing for HIV therapy [14]. Analysis of plasma levels of lopinavir one week after starting vaginal treatment, demonstrated that the drug was being absorbed systemically by this route of administration although levels of 10 ng/ml or less, showed that the concentration achieved was much lower than the 2.8–6.6 μg/ml previously reported for normal HIV therapy [14]. Nevertheless, these findings are consistent with the aim of treating cervical dysplasia whilst limiting systemic exposure to the drug.

The primary outcome was to assess tolerability and any adverse events associated with local application of this treatment to the cervix. Out of 23 women treated, no significant serious adverse indications were reported. Compliance was good and Lopimune was well tolerated with few side-effects and only nausea achieving statistical significance. Acceptability was also good, given that none of the subjects omitted a dose because of side-effects or because their partner wanted sexual intercourse. Moreover, 5 patients had intercourse during the treatment period and yet this did not appear to affect treatment outcome which suggests this may not be need to restricted. However, further study is clearly required to investigate this possibility.

A potential criticism of this study was the reliance on cytology for the pre-treatment and 4 week follow up diagnosis of underlying cervical dysplasia since it is well known that there is considerable variation (30–80%) in the accuracy of cytology between different centres throughout the world [26]. The Manchester-based cytology procedures, used in the current trial, were identical to those reported in the ARTISTIC study [27], which achieved >76% prediction of CIN2/3 from a diagnosis of HSIL.

Another potential criticism is the lack of a placebo control arm since CIN2/3 lesions can spontaneously regress if left untreated although there is a paucity of data regarding natural regression rates over a 3 month period with the majority of published work analysing this over 6 to 12 months or longer. However, a study, conducted by Alvarez et al, did report over 3 months and found that ~30% of CIN2/3 lesions regressed in this period [28] which was considerably higher than the 5% regression rate predicted by these authors. Moreover, they speculated that these higher than anticipated regression rates may have been due to biopsy related inflammatory changes [21] and yet, even allowing for similar spontaneous regression rates, this would not account for those observed in the current study.

Comparison of histopathology with cytology at 12 weeks post-treatment was mostly consistent although there were some differences. However, pathology still showed that ~60% of women had no detectable neoplasia at 12 weeks and others had reduced severity of disease (CIN1) indicating a combined positive response in ~77% of women. Most significantly these rates of regression are generally higher than would be predicted for untreated cases of high-risk HPV positive CIN2/3 [29].

Regarding the different HPV genotypes present, there was no significant correlation with response to treatment for any of those identified nor was there any obvious correlation with other socio-demographic factors such as age, type of contraception used, number of lifetime sexual partners or parity. Notably, none of the women in this study smoked.

How does the current treatment with Lopimune compare to other non-surgical treatments for cervical dysplasia? PDT has been extensively evaluated for this purpose with a large range of response rates ranging from 0–100% for CIN and 53·4–80% eradication of HPV [30]. Disadvantages are that PDT is physician applied with each sequential treatment typically taking several hours. Also systemic use of photosensitizers can cause problems with general skin sensitization to light. Topical application with the cytotoxic anti CMV drug Cidofovir has been used to treat CIN2/3 and showed ~60% clearance of CIN but did not eliminate HPV infection as assessed by the hybrid capture 2 assay [31]. Direct application of the immune activator Imiquimod to the cervix has been shown to have some effect against CIN and HPV infections both before and after LEEP although the treatment is continued for >8 weeks and the side effects can be quite severe [32,33]. A recent study of topical application of the cytotoxic drug 5FU to the cervix showed this to be >90% effective against CIN2 lesions in young women aged 18–29 although this was physician applied and the treatment period lasted 16 weeks [34]. A review of pharmacological approaches for the treatment of HPV related lesions was carried out by Bernard in 2004 [12] with the conclusion that none were generally recommended due to side effects and limited efficacy. Clearly, application of these modalities has improved in recent years although there are still many issues surrounding their implementation into clinical practice

The current study illustrates the potential of Lopimune to serve as an effective alternative to surgery for the treatment of HPV related cervical dysplasia although it is clear that the dose and regimen used in our study were sub-optimal and need further refinement. Evidence for this was that some women showed a transient improvement in HPV and/or cytology at 4 weeks but then went on to develop HPV positive abnormal cytology/pathology at 12 weeks (See Fig 2 E03, E13, E17). Advantages of this therapy are that; it can be self-applied,; it is relatively cheap (~£10.00 per patient for the current treatment protocol as purchased from Kenyan pharmacy in 2013); it is a non-cytotoxic FDA approved drug which does not target DNA and it has very good safety profile with a current license for the long-term systemic treatment of HIV infected pregnant women and children [35]. Furthermore, since HPV is DNA based and is more stable then RNA based HIV, it is less likely to develop resistance to drugs such as Lopimune which implies that, in principle, this treatment could be repeated many times.

In conclusion, this exploratory study illustrates the potential use of Lopimune as a self-administered treatment for HSIL with associated clearance of high risk HPV infections. It is very clear that more robust trials are needed to validate this new indication which would be particularly useful in low resource settings.

## Supporting Information

S1 Table Trend Analysis. (PDF)

S2 Table Patient Baseline and Outcome Data. (DOCX)

S1 Text LOTT Trial Protocol. (DOC)

S2 Text Structured Questionnaire. (DOCX)

S3 Text CRF Wks 1, 2, 4 and Months 2 & 3. (DOCX)

S4 Text CRF Wks 1 &2. (DOCX)

S5 Text KNH/UoN ERC Ethical Approval Sept 2015. (PDF)

## Abbreviations

ACN: Acetonitrile
ASC-H: Atypical squamous cells cannot exclude HSIL
ASCUS: Atypical squamous cells of undetermined significance
CIN: Cervical intraepithelial neoplasia
CMV: Cytomegalovirus
Depo: Depo provera
HAART: Highy active antiretroviral therapy
hCGIN: High grade cervical glandular cell intraepithelial neoplasia
HIV: Human immunodeficiency virus
HPV: Human papilloma virus
HSIL: High grade squamous intraepithelial lesion
HR: High risk
ICC: Invasive cervical cancer
IUD: Intrauterine device
KNH: Kenyatta National Hospital
LBC: Liquid based cytology
LEEP: Loop electrosurgical excision procedure
LLETZ: Large loop excision of the transformation zone
MSMS: Tandem Mass Spectrometer
ORF: Open reading frame
PCR: Polymerase chain reaction
PI: Protease inhibitor
VILI: Visual Inspection with Lugol’s iodine
VIA: Visual inspection with acetic acid
