# Supplementary material for: A Single-Arm, Proof-Of-Concept Trial of Lopimune (Lopinavir/Ritonavir) as a Treatment for HPV-Related Pre-Invasive Cervical Disease
Source: PLoS One. 2016 Jan 29;11(1):e0147917. doi: 10.1371/journal.pone.0147917 (PMC4732739; doi:10.1371/journal.pone.0147917)
Supplement: S2 Table — (DOCX) [file pone.0147917.s002.docx]

**Table 1: Patient baseline characteristics of women diagnosed with HSIL prior to treatment with Lopimune.**

| **E-No** | **Birth**  **Control** | **Parity** | **Age**  **(35.3Av)** | **HPV status** | | **Cytology** | |
| --- | --- | --- | --- | --- | --- | --- | --- |
|  |  |  |  | **Cervista** | **PCR** | **LBC** | **Conventional Smear** |
| E01 | Cond | 2 + 1 | 29 | M3 | 35 | sHSIL | ASC-H/AGC |
| E02 | TL | 2 + 0 | 39 | M1, 3 | 52 | sHSIL | HSIL |
| E03 | None | 2 + 0 | 35 | M3 | 16 | sHSIL | HSIL |
| E04 | IUD | 4 + 0 | 42 | M1 | 70 | mHSIL | HSIL |
| E06 | None | 2 + 0 | 29 | M2, 3 | 16, 39 | mHSIL | HSIL |
| E07 | IUD | 2 + 1 | 23 | M2, 3 | 18, 52 | mHSIL | LSIL |
| E08 | None | 2 + 0 | 27 | M3 | 52 | sHSIL | HSIL |
| E09 | IUD | 2 + 0 | 38 | M2 | 18 | sHSIL | HSIL |
| E10 | None | 2 + 0 | 37 | M2 | 45 | mHSIL | HSIL |
| E11 | None | 2 + 0 | 37 | M3 | 33, 58 | sHSIL | ASC-H |
| E12 | IUD | 2 + 0 | 41 | M2 | 18 | sHSIL | HSIL |
| E13 | None | 0 + 0 | 22 | M3 | 33, 58 | mHSIL | HSIL |
| E14 | None | 0 + 0 | 26 | M3 | 52 | sHSIL | HSIL |
| E15 | Depo | 3 + 0 | 40 | M2 | 68 | sHSIL | HSIL |
| E16 | Cond | 2 + 0 | 41 | M3 | 52 | sHSIL | HSIL |
| E17 | None | 2 + 0 | 38 | M1, 3 | 16, 31, 51 | sHSIL | HSIL |
| E18 | IUD | 2 + 0 | 36 | M3 | 33 | mHSIL | HSIL |
| E19 | PM | 7 + 0 | 69 | M3 | 58 | sHSIL | HSIL |
| E20 | Depo | 1 + 1 | 22 | M3 | 33, 58 | mHSIL | HSIL |
| E21 | None | 0 + 1 | 26 | M3 | 16 | sHSIL | HSIL |
| E22 | Depo | 6 + 0 | 43 | M3 | 16, 35 | mHSIL | HSIL |
| E23 | Jadelle | 2 + 0 | 27 | M3 | 16, 35 | mHSIL | HSIL |
| E29 | Depo | 5 + 0 | 47 | M3 | 16, 35 | mHSIL | HSIL |

**Key:** Patient Enrolment Number (E-No); Cervista Mix 1 (M1) (HPV 51, 56, 66, or 70); Cervista Mix 2 (M2) (HPV 18, 39, 45, 59, 68) ; Cervista Mix 3 (M3) (HPV 16, 31, 33, 35, 52, 58); Conventional smears were reported by MPO and LBCs by MS which showed no statistical difference by McNemar test (p>0.05). **Abbreviations Used**:- ASCUS: Atypical squamous cells of undetermined significance; ASC-H: Atypical squamous cells – cannot exclude HSIL; AGC: Atypical glandular cells; N: Negative; Depo: Depoprovera; TL: Tubal ligation; PM: Post menopausal; IUD: Intrauterine device; None of the women smoked;. m = Moderate dyskaryosis; s = Severe dyskaryosis.

**Table 2: Lopimune treatment outcome of women diagnosed with HSIL**

| **E-No** | **HPV**  **Type** | **1 Month**  **Cytology** | **1 Month**  **Cervista** | **3 Month**  **Cytology** | **3 Month**  **Cervista** | **3 Month**  **Path 1** | **3 Month**  **Path 2** |
| --- | --- | --- | --- | --- | --- | --- | --- |
| E01 | 35 | Normal | Negative | Normal | Positive | Normal | Normal |
| E02 | 52 | sHSIL | Negative | Normal | Negative | Normal | CIN2* |
| E03 | 16 | Normal | Positive | sHSIL | Positive | Normal | CIN1 & HCGIN* |
| E04 | 70 | Normal | Negative | Normal | Negative | Normal | Normal |
| E06 | 16, 39 | mHSIL | Positive | mHSIL | Positive | CIN1 | CIN1 just |
| E07 | 18, 52 | sHSIL | Positive | LSIL | Positive | CIN2 | CIN1–CIN2 |
| E08 | 52 | LSIL | Positive | Normal | Negative | Normal | CIN1* |
| E09 | 18 | sHSIL | Positive | ASCUS | Negative | Normal | Normal |
| E10 | 45 | mHSIL | Positive | LSIL | Positive | Normal | Normal |
| E11 | 35, 58 | Normal | Negative | Normal | Negative | Normal | Normal |
| E12 | 18 | Normal | Positive | Normal | Positive | Normal | Normal |
| E13 | 33, 58 | Normal | Negative | LSIL | Positive | CIN1 | CIN1 – CIN2* |
| E14 | 52 | Normal | Positive | Normal | Negative | Normal | Normal |
| E15 | 68 | Normal | Negative | Inadequate | Positive | Normal | Normal |
| E16 | 52 | Normal | Negative | Normal | Negative | Normal | Normal |
| E17 | 16, 31, 51 | Normal | Positive | mHSIL | Positive | CIN3 | CIN2 + CIN3 |
| E18 | 33 | Nromal | Negative | Normal | Negative | Normal | Normal |
| E19 | 58 | Inadequate | Inadequate | Normal | Negative | Not suitable | Not suitable |
| E20 | 33, 58 | LSIL | Positive | Normal | Positive | Normal | Normal |
| E21 | 16 | ASCUS | Positive | Normal | Negative | Normal | CIN1 just* |
| E22 | 16, 35 | Normal | Positive | Normal | Negative | Normal | Normal |
| E23 | 16, 35 | Normal | Negative | Normal | Negative | Normal | Normal |
| E29 | 16, 35 | Normal | Positive | sHSIL | Positive | Normal | CIN1* |
| **Totals** |  | **64% (14/22) Normal**  **14% (3/22) LSIL/ASCUS**  **Total Improved 78%** | **41% (9/22)**  **Negative** | **64% (14/22) Normal**  **18% (4/22) LSIL/ASCUS**  **Total Improved 82%** | **52% (12/23)**  **Negative** | **59% (13/22) Normal**  **18% (4/22) CIN1:**  **Total Improved 77%** | |

Cervista results are shown as % HPV negative. Cytology result totals are shown as % reverting to normal, % reverting to LSIL/ASCUS and the sum of these. Regarding pathology, where there was a differential diagnosis, a third pathologist reviewed the sections which confirmed the diagnosis of pathologist 2 at 3 months (Marked with *). The pathology totals shown are thus calculated from pathologist 2’s diagnosis and are shown as % reverting to normal, percentage with CIN1 and the sum of these.
