# Supplementary material for: A Single-Arm, Proof-Of-Concept Trial of Lopimune (Lopinavir/Ritonavir) as a Treatment for HPV-Related Pre-Invasive Cervical Disease
Source: PLoS One. 2016 Jan 29;11(1):e0147917. doi: 10.1371/journal.pone.0147917 (PMC4732739; doi:10.1371/journal.pone.0147917)
Supplement: S1 Text — (DOC) [file pone.0147917.s003.doc]

**Research Proposal**

Phase I Trial

**TITLE: Lopinavir as a Topical Treatment (LOTT) trial for HPV related cervical dysplasia in HIV negative women.**

**
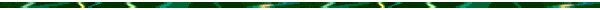
**

**PRINCIPAL INVESTIGATOR:**

**Dr Ian N. Hampson, PhD,**

Senior Lecturer, Department of Gynaecological Oncology, University of Manchester.

**SITE PRINCIPAL INVESTIGATOR:**

**Dr Maranga I.S.O,**

MBChB, PGD- STI/HIV, M.Med (Obs/Gynae), PhD (Gynae Oncology) candidate. Specialist Obstetrician /Gynaecologist and Senior Registrar.

**CO- PRINCIPAL INVESTIGATORS:**

**Dr Lynne Hampson, PhD,**

Lecturer, Department of Gynaecological Oncology, University of Manchester.

**Dr Peter Gichangi, MD, PhD**

Consultant Gynaecologist and Senior Lecturer, Departments of Anatomy and Gynaecology, University of Nairobi.

**Table of Contents**

Glossary of Abbreviations………………………………………………….…..........................3

Abstract for the study .............................................….......................4

Background ……………………………………………………..…………..…..…........................ 5

Objectives ………………………………………………………….……….…….…….......................5

Rationale ……………………………………………………………………...……….........................6

Literature Review ……….…………………….....................................................7

People and track record ....................................................................12

Methodology………………..……………………………………….………....………..................... 13

Data management and analysis ............................................................19

Ethics and research governance............................................................20

Ultimate benefits of the trial...............................................................23

References …………………………………………………………….….…………..........................24

**GLOSSARY OF ABBREVIATIONS.**

AOR : Adjusted Odds Ratio

ARR : Adjusted Relative Risk

ART : Antiretroviral Therapy

ARV : Antiretroviral

CI : Confidence Interval

CIN : Cervical Intraepithelial Neoplasia

CRFs : Case Report Form

ELISA : Enzyme Linked Immunosorbent Assay

H&E : Hematoxylene and Eosin Staining

HIV : Human Immunodeficiency Syndrome

HPE : Histopathological Examination

HPV : Human Papilloma Virus

HSIL : High Grade Squamous Intraepithelial Lesion

HVS : High vaginal swab

ICC : Invasive Cervical Cancer

KNH : Kenyatta National Hospital

LFTs : Liver Function Tests

LPV/r : Lopinavir/ritonavir

LSIL : Low Grade Squamous Intraepithelial Lesion

MCS : Microscopy, culture and sensitivity

OD : Odds Ratio

PCR : Polymerase Chain Reaction

PI : Protease Inhibitor

QPCR : Quantitative Polymerase Chain Reaction

RNA : Ribonucleic Acid

RR : Relative Risk

SIR : Standardized Incidence Ratio

SOPs : Standard operating procedures

STI/STD : Sexually Transmitted Illness/Disease

TBC : Total Blood Counts

U/E/C : Urea, Electrolytes & Creatinine

VCT : Voluntary Counseling and Testing (of HIV)

# ABSTRACT

We have previously shown that the orally administered anti HIV protease inhibitor lopinavir (Kaletra, Abbott Labs) has *in vitro* activity against the human papilloma virus (HPV) albeit at higher doses than can be achieved by oral dosing. We have since defined the activity range, specificity, molecular mechanism and site of action of this compound against HPV. We now propose to test the effectiveness of Lopinavir as a topical treatment for HPV-related pre-invasive disease of the cervix in HIV-ve Kenyan women.

Initially 600 women attending Kenyatta National Hospital in Nairobi will be screened for eligibility to enter the study and offered a variety of clinical services free of charge eg cervical smears, colposcopy, HIV test, HPV test, STI testing and if need be treatment *(Note:- Cervista HPV testing and ThinPrep cytology will be carried out in Manchester).* In addition, reimbursement of patient travel costs will also be offered. Our previous studies have predicted that this sample size will contain ~30 HIV negative women with cervical dysplasia (CIN II/III). These will randomly be divided into two equal groups (15 each).

One Lopinavir soft gel, will be applied vaginally to the cervix of half the patients once daily, while the other half will receive twice daily dosage; both groups for 2 weeks. These women will be examined every 2 days for adverse reactions and tolerability (Monday, Wednesday and Friday).

Before and immediately post-treatment, the cervix will be examined by colposcopy and a liquid based cytology (LBC) sample taken for HPV testing and cytology. The same procedures will be carried out at follow-up periods of; 1 & 3 months with HPV testing and ThinPrep cytology (Manchester) used to determine the extent of HPV related disease. During the course of study, any women identified with high-grade dysplasia will be offered conventional treatment/surgery within 3 months of diagnosis. Any patient diagnosed with carcinoma in situ or invasive cervical cancer during screening or trial period will be referred for immediate urgent intervention in the Gynaecology out-patient clinic (GOPC).

**Background**

Cervical cancer is the single largest cause of women's cancer mortality in Kenya where HPV related pre-cancerous cervical dysplasia is ~20 times more common in HIV+ve than in HIV-ve women. Furthermore, unlike HIV-ve women, HIV+ve women respond very poorly to conventional surgical treatment for cervical dysplasia. Moreover surgery is not widely available in Kenya which is economically poor with >60% of the population surviving on <$2.00/day. Thus it is clear that expensive HPV vaccines are currently not an option in this low resource setting. In any case, even if HPV vaccination were possible, there is evidence to indicate this will have reduced efficacy in HIV+ve women. What is needed is a simple, preferably self-applied treatment for HPV related cervical lesions that will work in both HIV-ve and HIV+ve women.

**Lay Summary**

We have previously shown that the main component of the drug Kaletra, normally given as tablets to treat HIV infection, may also be active against the human papilloma virus (HPV) which is the main cause of cervical cancer. However, this anti HPV activity requires a higher dose than can be achieved from orally administered tablets. Since this work was first published in 2006 we have now clarified exactly how Kaletra works against HPV. It is now our intention to conduct a clinical trial to test the effectiveness of Kaletra as a topical, self-applied treatment for HPV related pre-cancerous disease of the cervix in HIV-ve Kenyan women. It is intended to screen 600 women for early stage disease before cervical cancer develops. Based on our previous work, it is predicted that this population will have approximately 30 HIV –ve women with HPV-related cervical disease.

**BROAD OBJECTIVE:**

To evaluate the safety/ tolerability and potential effectiveness of vaginal Lopinavir soft gels as treatment for HPV mediated cervical disease in HIV negative patients.

**Specific Objectives:**

1. To determine the safety/ tolerability of vaginal Lopinavir soft gels as treatment for HPV mediated cervical disease in HIV negative patients.

2. To determine the potential effectiveness of Lopinavir soft gels as treatment for HPV mediated cervical disease in HIV negative patients.

**RATIONALE:**

***The Problem****:* Globally approximately 40 million people are infected with HIV with over 60% of these living in sub-Saharan Africa (UNAIDS, (2007)). Kenya is a sub-Saharan African country with almost 2 million people infected with HIV/AIDS of which 1.2 million (8.7%) are women (Kenya Demographic Health Survey, 2003). Additionally, the country lacks an effective public health system and therefore no structured cervical smear testing program is available. The combination of these factors with high poverty levels has invariably led to a high prevalence of cervical dysplasia and cancer (Gichangi P, 2002).

Cancer of cervix is the 3^rd^ leading cause of cancer deaths in women worldwide with global annual estimates at 452,000 new cases and more than 270,000 deaths (Parkin, 1999). Developing countries, such as Kenya, bear the bulk of the twin burdens of HIV/AIDS and cervical cancer. In reality this disease is the leading cause of cancer-related deaths among women in low resource countries and it is also the commonest female reproductive tract cancer in Kenya (Parkin, 1999). As a matter of fact it is the most common cancer in Kenya per se (Temmerman M, 1999) where this disease accounts for 18 to 23% of all cases of cancer diagnosed. The International Agency for Research on Cancer has also estimated the age-standardized incidence of cervical cancer in Kenya as 36.56 per 100,000 women (IARC., 2000) with prevalence rates of pre-cancerous cervical dysplasia ranging from 2.56% to 16.7% (Temmerman M, 1999).

HIV infection is known to be a significant risk factor for cervical cancer, with HIV positive women being approximately 20 times more likely to develop squamous intraepithelial lesions than HIV negative women (Wright TC, 1994, Parkin DM, 1999). It is significant that these observations have also been confirmed in Kenyan studies (Maranga O, 2011, Gichangi P, 2002, De Vuyst H, 2003). What is clear is that HIV, HPV and cervical cancer are epidemiologically linked whereby infection with one of these agents predisposes to infection with the other which can lead to the development of malignant disease.

It is also noteworthy that HIV/AIDS positive women respond poorly to convectional surgical approaches for cervical dysplasia which are otherwise effective in HIV negative women (Heard, 2004). Furthermore, there is also evidence that post-operative HIV shedding increases 10,000 fold in the cervico/vaginal fluid of HIV+ve women who have undergone surgery for cervical dysplasia which obviously increases the risk of viral transmission via sexual contact (Wright TC, 1994). Yet even with these limitations, surgery is neither widely nor readily available in Kenya where almost 60% of the population lives below the poverty line (i.e below US $2.00 per day) (International Monetary Fund, 2005). It's therefore evident that, with current pricing strategies, costly HPV vaccines are not a viable option in such low income countries. Moreover, even if HPV vaccination were possible, there is evidence to indicate this will have significantly reduced efficacy in HIV positive women (Ali-Risasi C, 2008 ). What is needed is a simple, non-surgical, low-cost, preferably self-applied treatment for HPV related cervical lesions that will work in both HIV-ve and HIV+ve women in sub Saharan Africa. We propose that topical treatment with the anti HIV protease inhibitor lopinavir could fulfil this need.

**LITERATURE REVIEW:**

***Supporting Work on the mode-of-action of Lopinavir against HPV;*** For a virus to successfully infect a target cell and achieve replication it must evade the host immune response and subvert many different cellular processes. It is interesting that different viruses, with very different tissue tropisms, have evolved very similar mechanisms to accomplish this goal. An example of this is provided by virus-induced inappropriate activation of the cellular ubiquitin-activated 26S proteasome whereby cellular proteins which are detrimental to the virus life cycle are degraded (Banks et al., 2003). One such protein is the cellular tumour suppressor p53 which is targeted by multiple viruses (Carmen Rivas et al., 2010). Thus it is significant that we have previously shown that the HIV protease inhibitor (PI) lopinavir can inactivate the ability of HPV16 E6 to induce the degradation of p53 in cultured cervical carcinoma cells and this effect is accompanied by apoptosis (Hampson et al., 2006). However, the concentration of lopinavir required for this activity was 15 - 25 µM which is approximately 15 - 20 times higher than can be achieved in cervico/vaginal fluid by oral dosing with this drug as part of highly active antiretroviral therapy (HAART) (Min et al., 2004).

In order to provide additional validation of the potential therapeutic effects of lopinavir against HPV, we have since carried out further investigations on the mode-of-action of this drug against HPV positive cells and much of this work was presented at the 25^th^ International Papilloma Virus Conference [18]. Since we had demonstrated that lopinavir stabilised p53 in the presence of HPV16 E6 (Hampson et al., 2006), it was decided to focus on proteomic analysis of lopinavir treated cells in order to identify any other cellular proteins, in addition to p53, which may be involved in the anti-HPV effects of this compound. Total protein extracts from

lopinavir and control DMSO treated SiHa cervical carcinoma cells were analysed by immuno-probing an antibody micro-array (Panorama Xpress 725, Sigma). Most notably this showed that the interferon-inducible antiviral protein Ribonuclease L (RNAse L) was up-regulated by this treatment and this result was validated by western blot (Fig. 1a) (Gavin Batman, 2011). It was also shown that lopinavir had no effect on the levels of RNAse L mRNA in these cells (Fig 1b) indicating this effect is likely due to inhibition of HPV mediated proteasomal of RNAse L protein (Gavin Batman, 2011).

**RNase L**


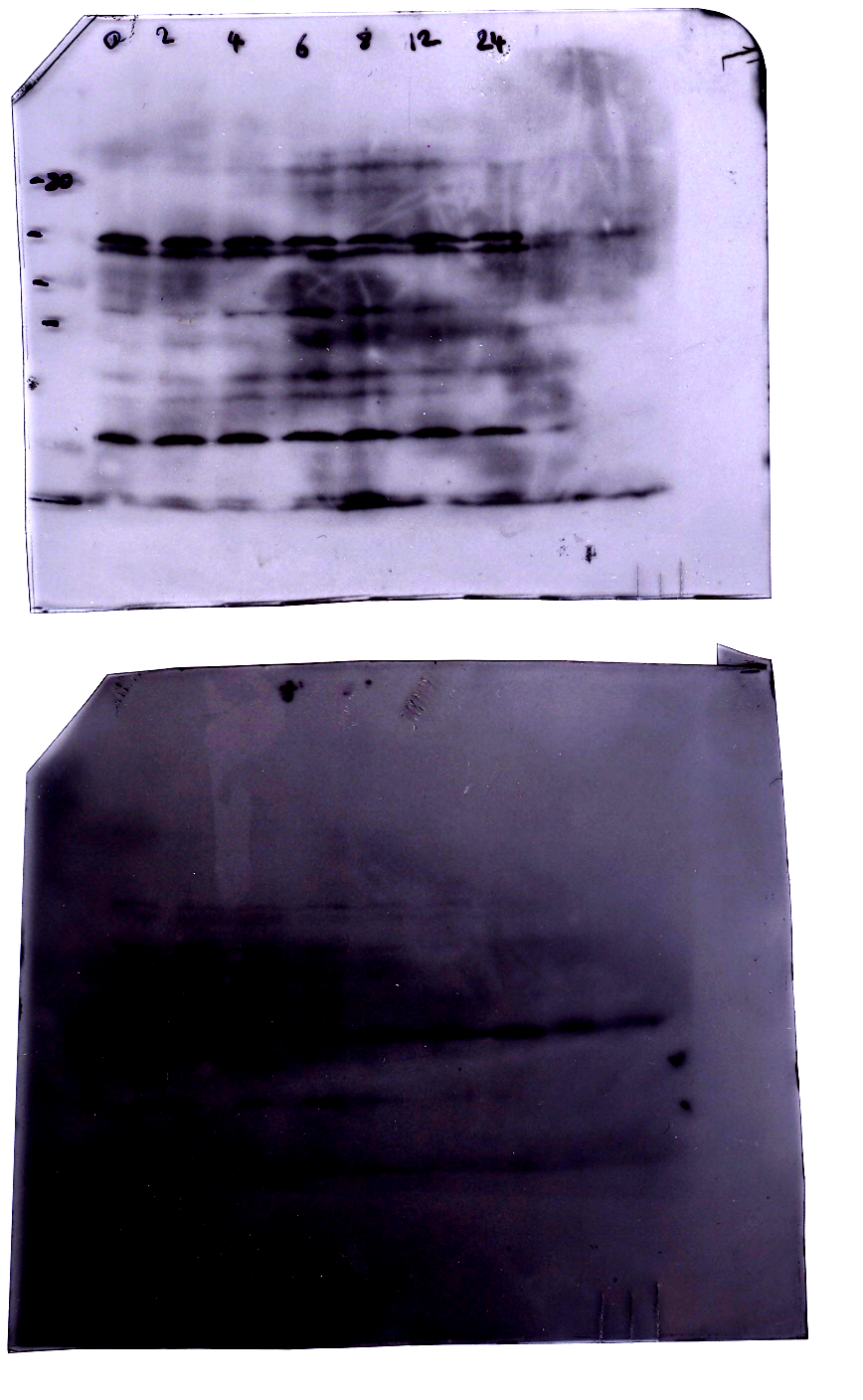

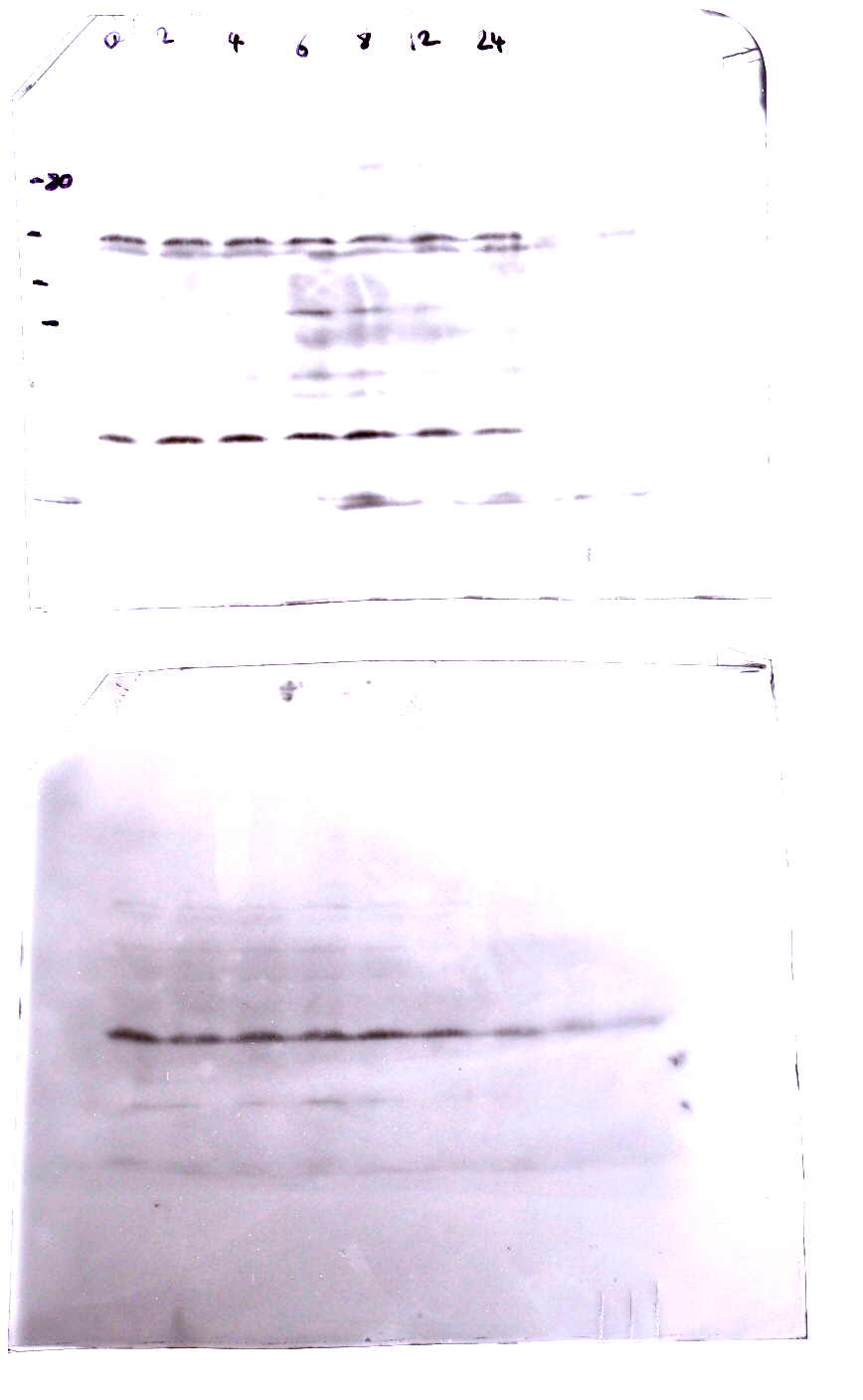


**0 2 4 6 8 12 24**

**GAPDH**

**hrs (25µM lopinavir)**


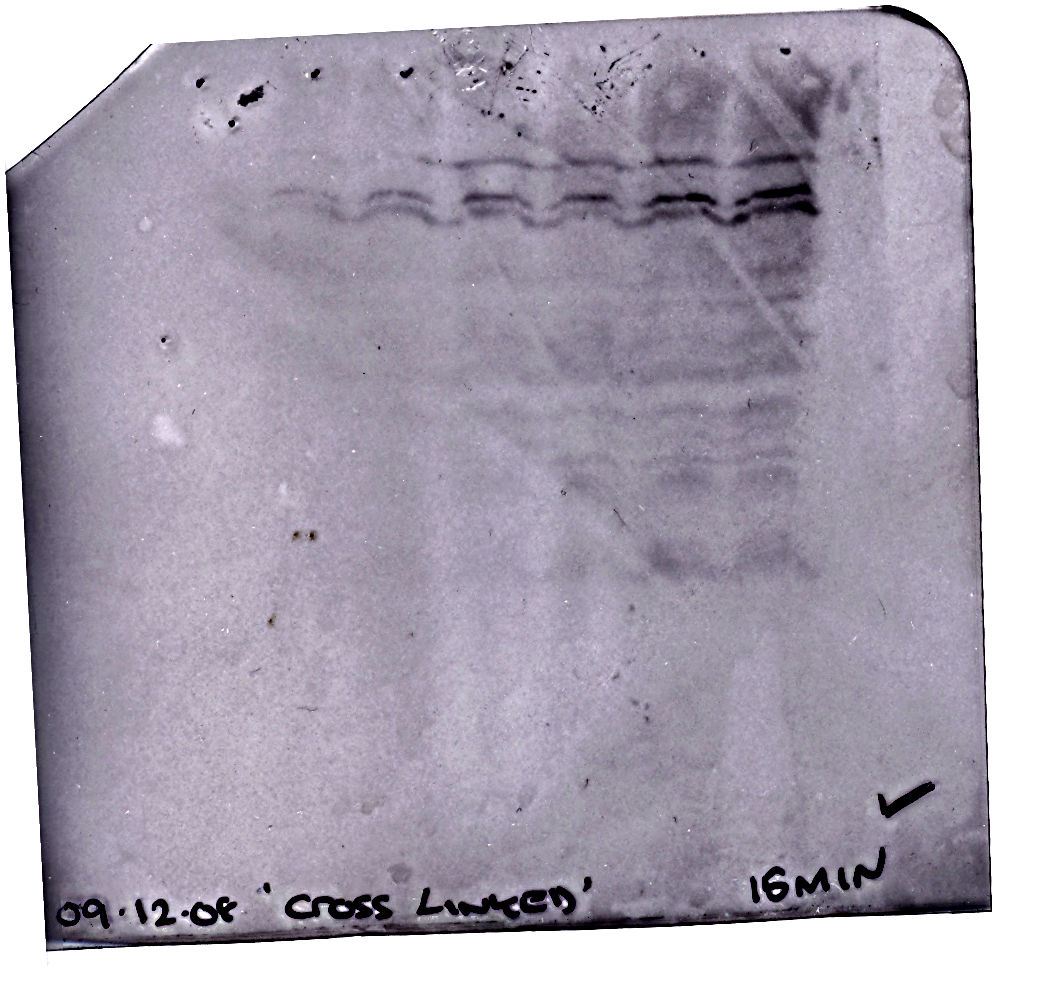


**5**

**Lopinavir (µM) (6hrs)**

**DMSO**


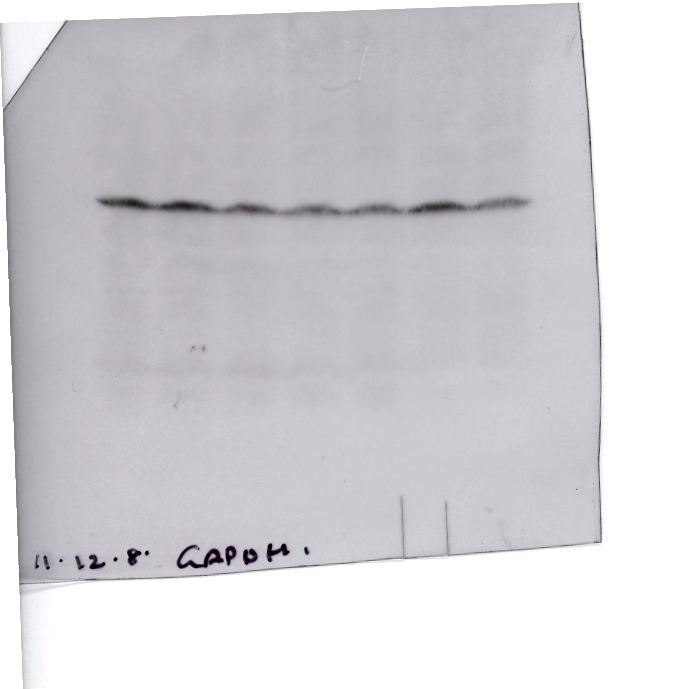


**RNase L**

**GAPDH**

**30**

**25**

**20**

**15**

**10**

**0**

**Figure 2**

b)

(a) – Time course western blot of lysates isolated from SiHa cells treated with 25µM lopinavir over a 24hour period. Demonstrates an increase in RNase L levels with a peak between six and eight hours before falling.

(b) – Dose response western blot of lysates from SiHa cells treated for 6 hours at the lopinavir concentrations shown. Demonstrates a clear dose response of RNase L levels to lopinavir concentrations. (Gavin Batman, 2011).

a)

RNAse L is an important component of antiviral cellular defence systems and it has also been implicated as a tumour suppressor (Silverman, 2007, Silverman, 2003). In order to clarify the role of this protein in the response of HPV positive cervical carcinoma cells to lopinavir we carried out time course and dose response analysis of SiHa cells treated with this compound (Gavin Batman, 2011). Figure 2 clearly shows that the RNAse L protein is up-regulated by lopinavir in a time and dose dependent manner. The marked up-regulation observed between 6 and 8 hours is rapidly followed by apoptosis at 24 hours which explains the observed drop in GAPDH and RNAse L signals (Gavin Batman, 2011).

In order to validate the role of RNAse L in the toxicity of lopinavir against HPV positive cervical carcinoma cells, transient siRNA silencing of RNAse L expression was carried out in SiHa cells exposed to Lopinavir (Gavin Batman, 2011). This clearly showed that silencing RNAse L in these cells increased their resistance to lopinavir supporting a role for this protein in the toxic effects of this compound.

All the experiments described so far have been carried out using HPV positive human cervical carcinoma cell lines. However, this is not a true representation of the intended target cell for this prospective new treatment since it is clear that HPV positive pre-cancerous cervical lesions are not malignant. With this in mind, it was decided to investigate the toxicity of lopinavir against HPV16 E6/E7 immortalised keratinocytes (Richard et al., 2010). In collaboration with Professor I. Zehbe (Lakehead University, Canada) control and E6/E7 expressing keratinocytes were exposed to a lopinavir dose range. Figure 4a clearly shows that E6/E7 expressing cells are selectively killed by treatment with increasing concentrations of lopinavir for 3 days whereas the control keratinocytes are unaffected by concentrations of lopinavir up to 25 µM. The results shown in Figure 4b are also

consistent with our previous data since they show that the RNAse L protein is up-regulated in E6/E7 expressing cells after 48 hours treatment with 25 µM Lopinavir (Gavin Batman, 2011). These observations support the hypothesis that lopinavir exhibits selective toxicity for HPV infected keratinocytes and suggest that RNAse L is involved in this effect (Gavin Batman, 2011). Furthermore they also indicate that this treatment has a good therapeutic index for HPV related pre-cancerous cervical disease. As a follow on to these experiments, it was decided to investigate the hypothesis that HPV might compromise the antiviral activity of RNAse L and lopinavir may reverse this effect. In order to address these issues we used the human HPV negative cervical carcinoma cell line C33A and the same cells which we had previously stably transfected with HPV16 E6 (Hampson et al., 2001). We found that C33A cells did not express RNAse L so C33A and C33AE6 cells were both transiently transfected with the full open reading frame of RNAse L cloned into the mammalian expression vector pReceiver-MO1 (Genecopoeia). This showed that C33AE6 cells tolerated ectopic expression of RNAse L whereas this proved to be extremely toxic in the parental C33A cells (Figure 5) indicating that the E6 protein may inhibit the antiviral activity of the RNAse L system in these cells (Gavin Batman, 2011). This begs the question, how does E6 suppress the activity of RNAse L? It is known that RNAse L has an endogenous so-called RNAse L inhibitor (RLI also called ABCE1 or HP68) which co-immunoprecipitates (CoIP's) with RNAse L (Bisbal et al., 1995). We immuno-precipitated RLI from transient RNAse L-transfected C33A and C33AE6 cells then probed this material with anti RNAse L antibody (Fig. 6). (Co-transfection with a β-galactosidase expression plasmid was used for normalisation of transfection efficiencies between C33A and C33AE6 cells) This repeatedly showed that E6 expressing cells have more RLI associated with RNAse L than the control C33A cells (Gavin Batman, 2011). Furthermore, analysis of the RLI/RNAse L CoIP product from RNase transfected C33AE6 cells treated with 25 µM lopinavir, showed that this caused a pronounced time-dependent drop in the amount of RNAse L associated with RLI (Fig. 6a) whereas the total level of RNAse L detected in these cells did not drop to the same extent (Fig. 6b) (Gavin Batman, 2011).

***Summary;*** Our data indicate that lopinavir up-regulates the expression of the RNAse L protein in full HPV genome containing cervical cancer cells and in E6/E7 immortalised keratinocytes (Figs 1, 2 & 4). Furthermore, lopinavir shows selective enhanced toxicity against the latter when compared to control cells. We also found that HPV16 E6 facilitates increased association between RNAse L and RLI which may protect E6 expressing cells from the apoptosis-inducing activity of RNAse L. Significantly lopinavir appears to reduce the amount of RNAse L that is bound to RLI in E6 expressing cells which may augment its antiviral activity.

One final point worth noting is that RLI is also known to interact with the HIV Gag protein and this interaction is essential for HIV capsid assembly (Zimmerman et al., 2002). Since our results showed lopinavir reduced the association between RNAse L and RLI, it is possible that lopinavir may also modulate the interaction of Gag with RLI. This would suggest a novel, hitherto unknown, mode-of-action of lopinavir against HIV.

**PEOPLE & TRACK RECORD:**

From 2003 - 2009 Dr's Ian N. (INH) and Lynne Hampson (LH) were both members of the International Atomic Energy (IAEA) Co-ordinated Research Project (CRP E33026) to improve the survival of HIV+ve women with cervical cancer. It was during this time that INH and LH discovered the potential anti-HPV properties of lopinavir and supervised/ directed all the previously described validation experiments. Indeed both INH and LH have a proven track record in the assessment of the off-target effects of antivirals (Donne et al., 2009a, Donne et al., 2009b, Donne et al., 2007) and other compounds (Hampson et al., 2009).

Dr Peter Gichangi (PG), INH and LH are currently joint supervisors of Dr Innocent Orora Maranga (IOM) who is predicted to gain his PhD in early 2012. IOM is currently a split-site student between the Universities of Manchester and Nairobi which has provided valuable previous experience as regards establishing a working relationship between the two institutions.

IOM will be primarily responsible for recruiting the patients and carrying out the proposed clinical trial in collaboration with PG. INH and LH will supervise the Manchester based laboratory investigations associated with the project.

**METHODOLOGY**

**Study Site/ Population:**

It is envisaged that the clinical and basic diagnostic aspects of this study will be carried out at Kenyatta National Hospital in Nairobi whereas the scientific aspects of the work that require access to specialist equipment, will be carried out in Manchester.

The study subjects will be recruited among patients attending Kenyatta National Hospital's Family Planning Clinic and Gynaecology Out-patient Clinics in Nairobi. Biological specimens will be analyzed at the Kenyatta Hospital's laboratories whilst others will be shipped to the University of Manchester, UK for advanced analytical techniques.

Kenyatta National Hospital is the largest teaching and referral hospital in the East and Central African region with a bed capacity of about 2000. It receives referrals from all over the country and also from the entire region. It is also the University of Nairobi's teaching hospital.

The new University of Manchester Gynaecological Oncology research laboratories are situated in the Central Manchester Foundation Trust St Mary's hospital. The laboratories were commissioned in May 2009 and are equipped with state-of-the art facilities. In addition they also provide easy access to Medical Virology, Cytology and the Manchester Interdisciplinary Biocentre which are all important components of this proposal. The Gynaecological Oncology research group currently consists of INH, LH, 3 x post doctoral staff and 9 x PhD students which ensure a lively and productive research environment.

**Study design:**

This is a Randomized Phase 1 Clinical Trial.

**Study Period:**

The estimated start date of the study is Feb/March 2012 and the study will run for 1 year. It is predicted that it will take 6 months to screen the required 600 women needed to produce approximately 30 HIV-ve patients with HPV related cervical dysplasia.

The Cervical Intraepithelial Neoplasia (CIN) classification will be used for pap smear slide interpretation.

**Sample Size:**

The study aims to initially screen 600 women; from whom approximately 30 will be predicted to be HIV-ve with CIN II/III cervical dysplasia. These 30 cases will be randomized into two groups of either once daily or twice daily Lopinavir and the effects of these treatments analysed during follow-up.

About 6% of the target study HIV negative female population are known to have cervical disease (Maranga O, 2011). Given that the current HIV infection prevalence in Nairobi is 8.8% (Kenya Demographic Health Survey, 2009) then 600 patient screening is likely to produce at least 30 HIV negative women with cervical disease:

8.8% x 600= 53 HIV positive patients

(600- 53) x 6% = 33 HIV negative patients with cervical disease.

**Inclusion criteria*:***

1. Women aged above 18 yrs.
2. Patients who freely agree to join the study after extensive information and counselling; must also give written informed consent.
3. Patients must be able to receive and understand verbal and written information about the study.
4. Patients ready and willing to comply with the study follow-up schedule.

**Exclusion criteria**:

1. Patients under 18 yrs of age.
2. Patients who don't fulfil the above inclusion criteria.
3. Patients with conditions in which blood sampling may increase risk of complications eg sickle cell disease.
4. Known HIV positive patients.
5. Patients who’re too ill to give informed consent.
6. Persons who it is concluded through clinical judgment by the investigator should not participate in the study eg anticipated poor study compliance.
7. Patients who have had prior surgical procedures on the cervix eg Cone biopsy, hysterectomies etc.
8. Patients with invasive cervical cancer or carcinoma in situ.

**DATA COLLECTION/ LABORATORY PROCEDURES**

[Abbreviations: LBC- Liquid Based Cytology, PSC- Patient Support Centre, CCC- Comprehensive Care Clinic (for HIV/AIDS patients), GOPC- Gynaecology Out-Patient Clinic, CIN- Cervical Intra-epithelial Lesion, ICC- Invasive Cervical Cancer, CIS- Carcinoma In Situ]

**Procedures/ Specimens to be taken:**

1. Blood samples
2. Pap smears /Liquid based Thinpreps
3. High vaginal swabs (HVS)
4. Colposcopy

**Tests to be done:**

1. HIV test
2. Standard pap smear
3. MCS- microscopy, culture and sensitivity for HVS specimens for STIs screening
4. Serum drug levels/ concentrations (lopinavir)
5. Full blood count
6. Urea & electrolytes
7. Liver Function Tests (LFTs)
8. Colposcopy/ biopsy

# Dose and regimen of Lopinavir (Lopimmune Soft-Gel Capsules – CIPLA Pharma)

# Two dosing regimens will be used:

# Lopimmune soft-gel pessary once daily (OD) for 14 days.

# Lopimmune soft-gel pessary twice daily (BD) for 14 days.

# During this time patients will be seen every 48 hrs for assessment of any adverse effects/ tolerability.

# Adverse effects/Side effects to be explored:

# Unusual local/ vaginal sensations, such as burning, tingling, or numbness.

# Dyspariunia

# Vaginitis/ cervicitis/ vulvitis

# Vaginal discharge

# Vaginal dryness

# Other known systemic adverse effects of oral Lopinavir.

**Schedule of patient assessment/Sample collection:**

- 1. The initial 600 study subjects screened will be asked to undergo an HIV test and a Pap smear.
  2. They will be seen after two weeks for Pap smear results. Those with normal cytology will be discharged from the study while those with abnormal cytology will be given a HPV test.
  3. Study subjects with abnormal cytology plus a positive HPV test will be entered into the trial and randomized into either the ONCE DAILY or TWICE DAILY Lopinavir groups.
  4. Case Report Forms (CRF's) will be filled in for every visit by the study doctor, and various follow-up tests will be carried out as illustrated below.

**Scheme for patient visits and sample collection during the study Trial (n=30)***.*

| **Time** | **Tests** |  |  |  |  |  |  |  |  |
| --- | --- | --- | --- | --- | --- | --- | --- | --- | --- |
|  | **U/E** | **LFT** | **TBC** | **Lop** | **HVS** | **Pap** | **LBC** | **HPV** | **Colp** |
| **T0** | X | X | X | - | X | X | X | X | X |
| **Wk 1** | X | X | X | X | - | - | - | - | - |
| **Wk 2** | X | X | X | X | - | - | - | - | - |
| **Wk 4** | X | X | X | X | X | X | X | X | X |
| **Mth 2** | - | - | - | - | - | - | - | - | - |
| **Mth 3** | - | - | - | X | X | X | X | X | X |

(Abbreviations: U /E/C = urea, electrolytes & Creatinine, LFTs = liver function tests; TBC = total blood counts; Lop = lopinavir serum level; HVS = High vaginal swab for STI; Pap = Pap smear; LBC = liquid based cytology Thinprep; HPV = Human Papilloma Virus test; Colp = Colpsocopy ).

During the baseline visit, 10-20 mls of whole blood will be taken for the necessary laboratory tests namely, haemogram, renal & liver function tests. Other tests to be carried out will be HVS (STI), Pap smear, LBC and colposcopy.

**Clinical data collection:**

After a signed and dated form has been obtained, the following data will be recorded and entered into a clinical record form (CRF):

1. Socio-demographic data which includes patient's age, marital status, parity, smoking & alcohol history, family history of cancer, co-morbid conditions, concomitant medications, history of STIs.

2. Haematological, immunological and biochemical tests

1. Pap smear/ HPV test and colposcopy findings

**Follow-up data**:

This will include:

1. Dates when Lopinavir was given.
2. Any adverse effects/ tolerability.
3. Infections noted eg candidiasis /STIs.
4. Other treatments given eg for STIs.
5. Status of the cervix before and after treatment with Lopinavir.
6. Response to treatment: regression, stable disease or progression of the cervical dysplasia.

***Data Storage:*** The data from this study will be electronically stored and this information carried in the Study Master File (SMF). The Investigator Study File will be used to store any copies of the study variables of interest.

***Suspected Unexpected Serious Adverse Reactions (SUSARs):*** Serious adverse reactions associated with sample collection eg during phlebotomy or pap smear collection will be considered unforeseen and will be reported to the sponsor within 24 hours of the event. Adverse effects associated with surgical procedures or progression of the underlying cervical disease will not be considered unexpected in the context of this trial.

***Sample collection and handling:*** All recruited patients will be given study numbers which will be used for all data collection/ laboratory processes. Patients' names will not appear anywhere else except in the coding data sheet to be kept safely by the Site Principal Investigator. All patient specimens will be processed according to the laboratory manual and Standard Operating Procedures (SOPs). After collection, samples will be immediately anonymised by assigning a unique study code and logged on to a paper log upon receipt into the laboratory as per SOPs. In particular, all samples will be labelled in non-water soluble black ink with the anonymised patient number, date and time of collection. LBC samples and cervical biopsy material will be sent to the University of Manchester where this will be used to carry out cytology, HPV tests and analysis of lopinavir associated bio-marker expression.

**Custodians of the samples will be:**

Kenya:- IOM and PG, Manchester:- INH and LH

**Biohazards and safety*:***

All the processes involved in the collection, handling and storage of tissues and blood specimens will be carried out by personnel who have adequate experience and have received training in the risks associated and safety requirements.

**Data Management and Analysis**

Data will be collected using well structured questionnaires/ CRFs. When complete, these will be kept in a safe place ready for the data entry and for the confidentiality of the patients’ details.

After cross checking the questionnaires for any missing entries, a data base will be designed in MS Access which allows the research to set controls and validation of the variables. On completion of the data entry exercise the data will be exported in a Statistical Package, SPSS – Version 16.0 for analysis (SPSS inc. Chicago, Illinois, USA).

The data will be presented in tables and figures where applicable. Parametric test will be used to examine if there’s any significant association between the continuous variables, while Pearson’s chi-square (non-parametric tests) will be used to establish the significant associations between the categorical and the qualitative variables. A logistic regression will be used to explore the significant variable in the modelling of the factors associated with HPV, cervical dysplasia and response to Lopinavir among these patients.

Comparison of means and proportion will be done using independent Student T-test and Fishers exact test where appropriate. Odds ratio (OR), adjusted OR (AOR) and the 95 % Confidence intervals (CI) will be used to measure strengths of associations. Relative risk (RR) in univariate analysis and adjusted relative risk (ARR) on multivariate analysis will also be computed. A p-value (two-tailed test) of 0.05 will be considered significant.

**ETHICS AND RESEARCH GOVERNANCE**

This research will be carried out in accordance with the ethical principles in the Declaration of Helsinki; ICH Good Clinical Practice; Human Tissue Act 2004; and other applicable regulatory pre-requisites. Prior to commencing this research, approval was sought from the Kenyatta National Hospital's Ethics and Research Boards (ERBs) which has been granted (See Approval Letter).

***Patient information and informed consent****:* Patients will be requested to give informed consent prior to joining the study after being explained clearly in the language they can understand the purpose of the study, the expected benefits and risks thereof. Refusal to participate in the study will not deny the patients appropriate management for their illness as per hospital protocol. Fundamentally, all patients will be at liberty to terminate their participation in the study without any consequences. All information from patients will be strictly confidential and refusal to participate in the study will not deny the patients appropriate management for their illness as per hospital protocol. Patients will be requested to give informed consent prior to joining the study after its purpose has been explained clearly in the language they can understand together with the expected benefits and risks thereof.

**Anticipated benefits to the patients are as follows:**

1. Free counselling and HIV testing
2. Prompt referral for HIV disease for relevant treatment and long-term follow-up with these results.
3. Free total blood count plus renal and liver function tests for recruited patients and advise where applicable
4. Free Pap smear and prompt treatment of abnormal smears within 3 months.
5. Free STI screening and treatment.
6. Free HPV screening and treatment follow-up.
7. Free colposcopy/biopsy for those with abnormal smears and referral where appropriate
8. Free STI screening and treatment of those diagnosed.
9. Transport re-imbursement for clinic attendance.

**Potential psychosocial risks/inconveniences to patients:**

Potential risks to study participants will be the known consequences of HIV positive results such as; violence, family rejection or neglect upon reporting of positive HIV results combined with psychological/ emotional trauma. These factors can be made worse by additional diagnosis of pre malignant or malignant cervical disease.

These potential risks will be reduced as much as possible by ensuring the availability of project staff to provide individualized face-to-face counselling to discuss patients' problems/concerns in the clinic even without an appointment. Also referral to the Patient Support Center (PSC) for further counselling and management of HIV infection will be carried out. Counselling to spouse and family members whom the index patient will refer the research team will also be done.

**Other anticipated risks/inconveniences to the patients are:**

1. Potentially frequent intimate physical/pelvic examinations: [privacy and patient dignity will be ensured at all times]
2. May undergo potentially painful procedures during collection of samples [in cases of colposcopy- this will be done under general anaesthesia and relevant analgesics will be prescribed/dispensed to minimize any pain thereafter]
3. Women may experience adverse effects from lopinavir treatment such as potential inflammation/vaginitis and vaginal dryness resulting into dyspariunia. These will be actively sought and treated promptly with subsequent stoppage of lopinavir in the case of moderate to severe reactions.

Basic clinical laboratory diagnostic procedures carried out in Nairobi (eg U/E test, Pap smears, STI tests etc) will be supervised by resident clinical pathology staff reporting directly to IOM and PG. More advanced analytical procedures carried out on material transported to Manchester (eg HPV test, LBC, lopinavir activated biomarker analysis) will be performed under strict Good Laboratory Practice (GLP) guidelines under the supervision of INH and LH.

***Patient confidentiality***: All information from patients will be handled with strict confidentiality. Having given informed consent, the patient will be assigned a unique trial number which will be recorded on a password protected computer database. All identifiable data will be kept in a different file only accessible to the Principal investigator or approved appointee. Maintenance of patient anonymity will be the investigator's responsibility.

***Indemnity and compensation:*** This trial is designed and spear-headed by academic investigators who hold no insurance against compensation claims for injury caused by participation in the study. However, local indemnity insurance for the study has been sourced locally in Kenya

**Data preservation for sharing**

As regards this aspect of the study, we will adopt a strict policy in line with internationally agreed GCP guidelines. Study data will be stored electronically in both Nairobi and Manchester. Information will be filed in the Study Master File (SMF). For safety reasons copies of individual CRF's will be made on site prior to any transportation and these will be stored in the Investigator Study File (ISF). Approvals relating to regulatory issues regarding secondary use of data will be in place prior to commencement of the study. Access to any of the study data set will be granted subject to all patient confidentiality issues being met and by approval of the LOTT study group.

**Public engagement with science**

The original work which led to this proposal was communicated to the lay public by means of the University of Manchester press office which led to a BBC Radio 4 interview with INH.

(<http://www.bbc.co.uk/radio4/womanshour/04/2006_35_thu.shtml>)

BBC on line news also carried the story:(<http://news.bbc.co.uk/1/hi/health/5282206.stm>)

In addition the article was carried by many newspapers throughout the world and is still featured on many websites eg <http://www.medicalnewstoday.com/articles/50660.php>

Most recently, we published our work on the mode of action of Lopinavir in HPV. This was publicised worldwide and can be found on various website e.g. We anticipate that communication to the public will be by press releases of this type which will be timed to coincide with publication of peer reviewed articles.

<http://www.youtube.com/watch?v=JVSfeYAqmUM>

<http://www.dailymail.co.uk/health/article-1383148/HIV-drug-prevent-cervical-cancer-killing-virus-causes-disease.html>

<http://www.ntn24.com/noticias/un-farmaco-para-tratar-el-sida-podria-prevenir-el-cancer-cervical-04779>

<http://articles.timesofindia.indiatimes.com/2011-05-04/health/29508198_1_hpv-cervical-cancer-cancer-cells>

**ULTIMATE BENEFITS OF THIS TRIAL**

It is hoped that the trial will provide valuable data to support the new use of Lopinavir pessaries as a treatment for HPV related cervical dysplasia in women. If the trial proves successful it is our intention to proceed to Phase II Trials whereby we will evaluate the effectiveness of lopinavir as both a treatment and a prophylactic for HPV related cervical disease in both HIV negative and positive women in Kenya and potentially South Africa. The proposed trial is thus the first stage in the prospective implementation of this treatment as a viable alternative to surgery for cervical disease in HIV negative and ultimately HIV positive women. If successful, it is clear that this would provide very significant benefits for many other countries which have a high incidence of cervical cancer. It is also true that a non-surgical treatment could potentially provide an alternative to the current watch and wait practice that is used to manage low-grade cervical disease in developed countries.

**REFERENCES:**

ALI-RISASI C, P. M., VAN RENTERGHEM L, 2008 Human papillomavirus genotype profile in Kinshasa, Democratic Republic of the Congo: implications for vaccination.*]. Med Trop (Mars).* Dec; 68**,** 617-20.

BANKS, L., PIM, D. & THOMAS, M. 2003. Viruses and the 26S proteasome: hacking into destruction. *Trends Biochem Sci,* 28**,** 452-9.

BISBAL, C., MARTINAND, C., SILHOL, M., LEBLEU, B. & SALEHZADA, T. 1995. Cloning and characterization of a RNAse L inhibitor. A new component of the interferon-regulated 2-5A pathway. *J Biol Chem,* 270**,** 13308-17.

CARMEN RIVAS, STUART A. AARONSON & MUNOZ-FONTELA., A. C. 2010. Dual Role of p53 in Innate Antiviral Immunity. *Viruses,* 2,**,** 298-313.

DE VUYST H, S. S., VAN RENTERGHEM L, ET AL. 2003. Distribution of Human Papillomavirus in a Family Planning Population in Nairobi, Kenya. *Sex Transm Dis* 30**,** 137-142.

DONNE, A. J., HAMPSON, L., HE, X. T., DAY, P. J., SALWAY, F., ROTHERA, M. P., HOMER, J. J. & HAMPSON, I. N. 2009a. Potential risk factors associated with the use of cidofovir to treat benign human papillomavirus-related disease. *Antivir Ther,* 14**,** 939-52.

DONNE, A. J., HAMPSON, L., HE, X. T., ROTHERA, M. P., HOMER, J. J. & HAMPSON, I. N. 2007. Effects of cidofovir on a novel cell-based test system for recurrent respiratory papillomatosis. *Head Neck,* 29**,** 741-50.

DONNE, A. J., HAMPSON, L., HE, X. T., ROTHERA, M. P., HOMER, J. J. & HAMPSON, I. N. 2009b. Cidofovir induces an increase in levels of low-risk and high-risk HPV E6. *Head Neck,* 31**,** 893-901.

BATMAN G, Oliver AW., ZEHBE I,RICHARD C, HAMPSON L, HAMPSON IN 2011. Lopinavir Up-rregulates expression of the Antiviral Protein ribonuclease L in Human Papilloma Virus- positive cervical cancer cells. *Antivir Ther,,* 16.

GICHANGI P, D. V. H., ESTAMBALE B, ROGO K, BWAYO J, TEMMERMAN M. 2002. HIV and cervical cancer in Kenya. *Int J Gynaecol Obstet.,* 76**,** 55-63.

HAMPSON, L., EL HADY, E. S., MOORE, J. V., KITCHENER, H. & HAMPSON, I. N. 2001. The HPV16 E6 and E7 proteins and the radiation resistance of cervical carcinoma. *Faseb J,* 15**,** 1445-7.

HAMPSON, L., HE, X. T., OLIVER, A. W., HADFIELD, J. A., KEMP, T., BUTLER, J., MCGOWN, A., KITCHENER, H. C. & HAMPSON, I. N. 2009. Analogues of Y27632 increase gap junction communication and suppress the formation of transformed NIH3T3 colonies. *Br J Cancer,* 101**,** 829-39.

HAMPSON, L., KITCHENER, H. C. & HAMPSON, I. N. 2006. Specific HIV protease inhibitors inhibit the ability of HPV16 E6 to degrade p53 and selectively kill E6-dependent cervical carcinoma cells in vitro. *Antivir Ther,* 11**,** 813-25.

HEARD, I., J.M. PALEFSKY, AND M.D. KAZATCHKINE, 2004. The impact of HIV antiviral therapy on human papillomavirus (HPV) infections and HPV-related diseases. *Antivir Ther,,* 9 13-22,.

IARC. 2000. Cancer Incidence, Mortality and Prevalence worldwide. *IARC Globocan 2000 Database*.

INTERNATIONAL MONETARY FUND, I. 2005. Kenya: Poverty Reduction Strategy Paper. *IMF Country Report,* No. 05/11.

KENYA DEMOGRAPHIC HEALTH SURVEY, K. 2003. Key Findings.

KENYA DEMOGRAPHIC HEALTH SURVEY, K. 2009. Key Findings.

MARANGA O, H. L., KITCHENER H, HAMPSON I, 2011. Prevalence of cervical dysplasia in HIV/AIDS populations in Kenya. *Manuscript*.

MIN, S. S., CORBETT, A. H., REZK, N., CU-UVIN, S., FISCUS, S. A., PETCH, L., COHEN, M. S. & KASHUBA, A. D. 2004. Protease inhibitor and nonnucleoside reverse transcriptase inhibitor concentrations in the genital tract of HIV-1-infected women. *J Acquir Immune Defic Syndr,* 37**,** 1577-80.

PARKIN, D. M., P. PISANI, AND J. FERLAY 1999. Estimates of the worldwide incidence of 25 major cancers. *Int J Cancer,* 80**,** 827-41,.

PARKIN DM, W. H., NAMBOOZE S, WABWIRE-MANGEN F. 1999. AIDS-related cancers in Africa: maturation of the epidemic in Uganda. *Aids,* 13**,** 2563-70.

RICHARD, C., LANNER, C., NARYZHNY, S. N., SHERMAN, L., LEE, H., LAMBERT, P. F. & ZEHBE, I. 2010. The immortalizing and transforming ability of two common human papillomavirus 16 E6 variants with different prevalences in cervical cancer. *Oncogene*.

SILVERMAN, R. H. 2003. Implications for RNase L in prostate cancer biology. *Biochemistry,* 42**,** 1805-12.

SILVERMAN, R. H. 2007. A scientific journey through the 2-5A/RNase L system. *Cytokine Growth Factor Rev,* 18**,** 381-8.

TEMMERMAN M, T. M., KIDULA N, CLAEYS P, MUCHIRI L, QUINT W. 1999. Risk factors for human papillomavirus and cervical precancerous lesions, and the role of concurrent HIV-1 infection. . *Int J Gynaecol Obstet.,* 65**,** 171-81.

UNAIDS (2007). Uniting the world against AIDS. Global HIVAIDS estimates. . *Un Report.* .

WRIGHT TC, J., ELLERBROCK TV, CHIASSON MA, VAN DEVANTER N, SUN XW. 1994. Cervical intraepithelial neoplasia in women infected with human immunodeficiency virus: prevalence, risk factors, and validity of Papanicolaou smears; New York Cervical Disease Study. *Obstet Gynecol,* 84:**,** 591-7.

ZIMMERMAN, C., KLEIN, K. C., KISER, P. K., SINGH, A. R., FIRESTEIN, B. L., RIBA, S. C. & LINGAPPA, J. R. 2002. Identification of a host protein essential for assembly of immature HIV-1 capsids. *Nature,* 415**,** 88-92.
