# Supplementary material for: A Single-Arm, Proof-Of-Concept Trial of Lopimune (Lopinavir/Ritonavir) as a Treatment for HPV-Related Pre-Invasive Cervical Disease
Source: PLoS One. 2016 Jan 29;11(1):e0147917. doi: 10.1371/journal.pone.0147917 (PMC4732739; doi:10.1371/journal.pone.0147917)
Supplement: S2 Text — (DOCX) [file pone.0147917.s004.docx]

**QUESTIONNAIRE:**
