# Supplementary material for: A Single-Arm, Proof-Of-Concept Trial of Lopimune (Lopinavir/Ritonavir) as a Treatment for HPV-Related Pre-Invasive Cervical Disease
Source: PLoS One. 2016 Jan 29;11(1):e0147917. doi: 10.1371/journal.pone.0147917 (PMC4732739; doi:10.1371/journal.pone.0147917)
Supplement: Supplementary file 5 [file pone.0147917.s005.docx]

APPENDIX VII: CRF: [For Visits: Wks 1, 2, 4 and Months 2 &3]

Questionnaire for Phase 1 Clinical Trial on Prospective use of lopinavir as a topical treatment for HPV infections in HIV negative women

1. Personal Number [ ]
2. Have you experienced any problems since your last visit?
   1. Yes [ ]
   2. No [ ]
3. If yes, what complaints have you felt?________________________
   ____
4. Specifically, have you experienced any of the following symptoms?
   a. Abnormal vaginal discharge
   b. Vaginal dryness
   c. Painful intercourse
   d. Vaginal itch/ irritation
   e. Vaginal bleeding/ spotting
   f. Any other genital symptoms ________________________
5. Have you also experienced any of these symptoms?
   a. Dizziness
   b. Light-headedness/ headache
   c. Fainting
   d. Sensation of abnormal heartbeats/ palpitation
   e. Decreased appetite
   f. Yellowing of skin/ eyes
   g. Pale stools
   h. Nausea
   i. Vomiting
   j. Abdominal Pain/stomach pains
   k. Stomach upsets
   l. Feeling weak
   m. Diarrhoea
6. What of these symptoms?
   a. Itchy skin
   b. skin rashes
   c. Increased thirst
   d. Increased frequency of urination
   e. Problems with vision
   f. Chest pains
7. Are you taking any other medication at the moment?
   1. Yes [ ]
   2. No [ ]

   If yes, which drugs are you on?
   a. antibiotics
   b. Painkillers
   c. Oral contraceptive pills
   d. Anti-seizure/ and-epileptic drugs

KENYATTA NATIONAL HOSPITAL APPROVED 25 NOV 2011 ETHICS & RESEARCH COMMITTEE

e. Steroids
f. Anti-asthma drugs
g. Anti-TB drugs
h. Anti-migraine drugs
i. Anti-diabetics
j. Anti-hypertensives
k. Not sure what they're for
l. In each case above please specify individual drugs.

8. Have you experienced any improvement of symptoms since taking this drug [lopinavir]?

a. Yes [ ]
b. No [ ]
c. Didn't have any symptoms

If yes, please state which symptoms have improved______________________
____

KENYATTA NATIONAL HOSPITAL
APPROVED
25 NOV 2011
ETHICS & RESEARCH COMMITTEE
