# Supplementary material for: A Single-Arm, Proof-Of-Concept Trial of Lopimune (Lopinavir/Ritonavir) as a Treatment for HPV-Related Pre-Invasive Cervical Disease
Source: PLoS One. 2016 Jan 29;11(1):e0147917. doi: 10.1371/journal.pone.0147917 (PMC4732739; doi:10.1371/journal.pone.0147917)
Supplement: Supplementary file 6 [file pone.0147917.s006.docx]

KENYATTA NATIONAL HOSPITAL
APPROVED
25 NOV 2011
ETHICS & RESEARCH COMMITTEE

APPENDIX VIII: CRF: [For Visits: Wks 1 & 2]

Questionnaire for Phase 1 Clinical Trial on Prospective use of lopinavir as a topical treatment for HPV infections in HIV negative women

1. Personal Number
2. How many times do you insert the vaginal Lopinavir? a. Once a day [ ]
   b. Twice a day [ ]
3. Since you started taking this treatment, have you missed a dose? a. Yes [ ]
   b. No [ ] Go to Q 6.
4. If yes, how many times: a. Once [ ]
   b. Twice [ ]
   c. Thrice [ ]
   d. Four times [ ]
   e. Five times [ ]
   f. More than 5x [ ]
   g. Other; kindly specify ________________ [ ]
   ____
5. If yes, what are the reasons for missing taking the drug?
   a. I simply forgot [ ]
   b. Because of side effects (reactions). Please specify which______[ ]
   ____
   c. I feel inconvenienced [ ]
   d. My partner wanted sex [ ]
   e. I feel uncomfortable inserting [ ]
   f. Due to the staining of my pants [ ]
   g. Other reasons: Kindly specify ________________________
6. Have you had sex since starting this treatment?
   a. Yes [ ]
   b. No [ ] End of Questionnaire
7. If yes, how many times? a. Once [ ]
   b. Twice [ ]
   c. Thrice [ ]
   d. Four times [ ]
   e. Five times [ ]
   f. More than 5x [ ]
   g. Daily [ ]
   g. Other; kindly specify ________________ [ ]
   ____
