# Supplementary material for: A Single-Arm, Proof-Of-Concept Trial of Lopimune (Lopinavir/Ritonavir) as a Treatment for HPV-Related Pre-Invasive Cervical Disease
Source: PLoS One. 2016 Jan 29;11(1):e0147917. doi: 10.1371/journal.pone.0147917 (PMC4732739; doi:10.1371/journal.pone.0147917)
Supplement: S5 Text — (PDF) [file pone.0147917.s007.pdf]

UNIVERSITY OF NAIROBI
COLLEGE OF HEALTH SCIENCES
P O BOX 19676 Code 00202
Telegrams: varsity
(254-020) 2726300 Ext 44355

KNH/UON-ERC
Email: uonknh_erc@uonbi.ac.ke
Website: www.uonbi.ac.ke
Link: www.uonbi.ac.ke/activities/KNHUoN

KENYATTA NATIONAL HOSPITAL
P O BOX 20723 Code 00202
Tel: 726300-9
Fax: 725272
Telegrams: MEDSUP, Nairobi

Ref: KNH-ERC/ 01/PUB/6

3rd September 2015

Prof. Peter Gichang/Dr.Orora Maranga
The Lopinavir Clinical Trial Team
Dept.of Reproductive Health/Anatomy
UoN/KNH

Dear Prof.Gichangi & Dr. Maranga

Re: Authority to present for Publication a Manuscript titled "A pragmatic proof-of-concept trial of Lopimune(lopinavir/ritonavir) as a treatment for HPV-related pre-invasive cervical disease"

Refer to your communication of 3rd August 2015.

The KNH/UoN-Ethics and Research Committee acknowledges receipt of the above referenced manuscript submitted for institutional administrative/ethical vetting for suitability for presentation for possible publication.
The committee observes that this manuscript is the outcome of the implementation of the ERC approved clinical Trial "A Lopinavir as a Tropical Treatment for HPV-related cervical dysplasia in HIV negative women"(P273/05/2011).

Further the committee observes that overall the manuscript is publishable and no major ethical issues are anticipated from the disseminated information. However you are required to note and address the following issues:

i. Review the abbreviations and ensure that they tally.
ii. Expand on the results from the pre-clinical studies (page 5)
iii. KNH/UoN-ERC(Kenyatta National Hospital and University of Nairobi Ethics and Research Committee is the correct name of the approving body. (See page 5)
iv. Harmonise the venues or sites of recruitment in the abstract and methods(page 5).
v. Watch out for the placement of punctuations in some sentences (proof read for grammar).
vi. Page 6 refers to supplementary documents. It is not clear which ones they are and their significance.
vii. Do you need to state MPO or MD in a manuscript.
viii. Specify which 'bloods and health checks" that were done.
ix. Advised to change numbering from E to another letter since E can be confused in HPV results.
x. Spell out the serious adverse reactions that were expected "life threatening events e.g......"
xi. Make sure that you acknowledge the funding agency/agencies if any. (page 16)>

Kindly provide a copy of the final published manuscript to the ERC.

Yours sincerely

PROF. A.N. GUANTAI
CHAIRPERSON, KNH/UON-ERC

c.c. The Principal, College of Health Sciences, UoN
The Deputy Director CS
